# An Enhanced Dynamic Model of a Spatial Parallel Mechanism Receiving Direct Constraints from the Base at Two Point-Contact Higher Kinematic Pairs

**DOI:** 10.3390/biomimetics10070437

**Published:** 2025-07-03

**Authors:** Chen Cheng, Xiaojing Yuan, Yenan Li

**Affiliations:** Lab of Mechatronics, Rocket Force University of Engineering, Xi’an 710025, China

**Keywords:** Euler parameters, inverse dynamics, friction effect, spatial parallel mechanism, direct constraints from the base

## Abstract

In this paper, a biologically congruent parallel mechanism (PM) inspired by the masticatory system of human beings has been proposed to recreate complete chewing behaviours in three-dimensional space. The mechanism is featured by direct constraints from the base (DCFB) to its end effector at two higher kinematic pairs (HKPs), which greatly raise its topological complexity. Meanwhile, friction effects occur at HKPs and actuators, causing wear and then reducing motion accuracy. Regarding these, an inverse dynamic model that can raise the computational efficiency and the modelling fidelity is proposed, being prepared to be applied to realise accurate real-time motion and/or force control. In it, Euler parameters are employed to express the motions of the constrained end effector, and Newton–Euler’s law is applied, which can conveniently incorporate friction effects at both HKPs and actuators into the dynamic model. Numerical results show that the time consumption of the model using Euler parameters is only approximately 23% of that of the model using Euler angles, and friction effects significantly increase the model’s nonlinearity. Further, from the comparison between the models of the target PM and its counterpart free of DCFB, these constraints sharply raise the modelling complexity in terms of the transformation between Euler parameters and Euler angles in the end effector and the computational cost of inverse dynamics.

## 1. Introduction

Mastication is a complex process in ${\mathbb{R}}^{3}$, where the foods are chewed to smaller and softer boluses that can be swallowed safely [1]. The food industry has a strong curiosity in this process, since exploring the human-food interaction is beneficial for evaluating food texture properties so as to develop favourable, healthy, and appealing food products. Currently, machines used to assess food textures in vitro only simply compress the test foods in one dimension, which is far away from the real complicated chewing motions of human beings in ${\mathbb{R}}^{3}$. Recruiting healthy volunteers is associated with long-time consumption and high expenditure, and the results are not objective. Thereby, robotic devices that can accurately replicate complete masticatory behaviours in terms of chewing forces and motions in ${\mathbb{R}}^{3}$ are greatly needed. With their assistance, newly developed food samples can be chewed in a human-like manner, then food textures can be analysed reliably and efficiently. To this end, it is very natural to design a robotic mechanism by mimicking the muscle-skeleton biostructure of the masticatory system: its end effector is the moving mandible, the base is the fixed maxilla, and six revolute-spherical-spherical (RSS) parallel linkages working synchronously are the major chewing muscles, namely, the temporalis, the masseter, and the pterygoid [2]. The underlined letter R indicates it is the active joint. Left and right direct constraints from the base (DCFB) to the end effector form two higher kinematic pairs (HKPs), playing the role of the crucial temporomandibular joints (TMJs). As a result, from the viewpoint of mechanism, the designed prototype is a spatial parallel mechanism (PM) receiving DCFB to the end effector. These constraints evidently render this mechanism different from others, since PMs in general do not have this sort of constraints. They increase the topological complexity, bringing two parasitic motions and two redundant actuations simultaneously [2]. It is fundamental to stress that theoretically, two kinematic chains in the mechanism can be taken out to reduce its topological complexity; then it would have no actuation redundancy. Nonetheless, the biological features of the chewing system in terms of the roles of primary chewing muscles and TMJs cannot be explored adequately via this simpler mechanism, and the potential engineering applications could be limited. Hence, the bio-inspired mechanism under study is designed as faithfully as we can.

Due to the closed-loops, PMs are superior to their serial counterparts in terms of larger load carrying capacity [3], better motion accuracy [4,5], and lower moving inertias [6,7], even if their singularity problems are more complex and the workspace is smaller. Thus, they can exert these strengths in the domains where their desirable features are greatly needed [8,9], e.g., machine tools [10,11,12,13,14], fast pick-and-place manipulators [15], haptic devices [16], pointing devices [17], and physical human-robot interaction [18,19].

Before the designed mechanism is employed to evaluate newly developed foods in the food industry, there are two concerns about its practical applications. The first is that, due to the DCFB, parasitic motion variables are strongly coupled by translational and rotational degrees of freedom (DOFs). They have complicated trigonometric functions as in Equation (5) of [20], where *XYZ* Euler angles were used to characterise rotations of the end effector. These complex and lengthy equations considerably increase the computational time in dynamic models, being not in favour of real-time model-based controller design. Thus, more efficient alternatives to Euler angles are sought to reduce the computational cost. In the literature, Euler parameters, i.e., unit quaternions, deeply attract our interest. From the Euler theorem, the orientation of a rigid body can be defined by its rotation around an axis by an angle of rotation *θ* at any instant of time [21]. Thereby, these parameters are stated as

(1) $$\begin{array}{ccc} e={\left[\begin{array}{cc} e_{0} & e^{T} \end{array}\right]}^{T}, & e_{0}=\cos\frac{\theta }{2}, & e=u\cdot \sin\frac{\theta }{2} \end{array}$$

where **u** is the unit vector around which the rotation of the body occurs, and its three elements are the projections along the orthogonal axes of the inertia frame. Although four parameters are used to describe rotations in ${\mathbb{R}}^{3}$, indicating they are not completely independent and that one constraint equation is needed; this cumbersome feature is more than compensated for by their desirable merits as:

1. Any orientation of a body in ${\mathbb{R}}^{3}$ can be defined satisfactorily since there are no inherent singularity problems.
2. Kinematic equations associated with them have purely algebraic operators and are free of trigonometric functions, as with Euler angles, being computationally more efficient. As a result, they are easy to implement in a computer program in simpler and more compact manners.

Regarding these, they are adopted to describe the motions of the end effector of the mechanism under study to enhance the computational efficiency. From the literature, Euler parameters have been widely applied in many arenas due to their advantages. For instance, a general Euler parameter-based dynamic model was built by the Lagrange formulation for mechanical systems [22]. Many useful and interesting identities between Euler parameters and their derivatives were presented in [21], based on which the dynamic model can be more compact and efficient [23]. An attitude regulator for an arbitrary rigid body was designed with these parameters in [24], leading to a linear feedback law, where neither linearisation nor simplification assumptions such as small angular rates or attitude error angles were made. This presentation avoids nonlinear equations of motion (EOMs) and a nonlinear feedback law associated with Cayley–Rodrigues parameters. Euler parameters were utilised to model the end effector rotation errors in resolved rate and acceleration control of serial manipulators in [25]. The formulation considerably simplifies the stability analysis of orientation error equations. In [26], their utilisation in a spherical joint avoided the introduction of three virtual orthogonal revolute joints with zero length of intermediate links in the general manner, which raised the sizes of associated matrices in the EOMs and finally the computational burden. The dynamic modelling of a spatial RSSR serial mechanism showed that the CPU time has a 10% improvement over the virtual revolute joints method, and less computer memory is required. To address the photogrammetric problem, a closed-form solution was provided in [27], where unit quaternions were used to simplify the derivation of the solution.

As far as their applications in PMs are concerned, in [28], based on the number of constant zero components of Euler parameters, they were classified into 15 cases, and their kinematic interpretation was presented. Then, the orientation of the end effector of a 3-DOF 3-RER PM with orthogonal platforms was represented using Euler parameters effectively. Letters R and E denote revolute joint and planar joint, respectively. In [29], the above-mentioned 15 cases of classification were used again, then Euler parameters were employed together with Euler angles, algebraic geometry, and axodes to effectively identify and compare distinct continuous motion characteristics of three 2-DOF pointing mechanisms, i.e., a gimbal structure, a 1-RR&2-RRR spherical PM, and an Omni-Wrist III. A systematic classification of a 3-RER PM based on the type/number of operation modes varying with link parameters was presented in [30]. Euler parameters are found to be very useful for identifying the motion characteristics of the end effector.

Apart from the computational cost, the second concern in the designed mechanism is that, in practical experiments of its prototype, at the direct constraints from the base to the end effector, wear appears due to friction effects, further introducing clearances and reducing motion accuracy. Additionally, friction exists in the brushes, rotors, and bearings inside the actuators, but it is not desirable to disassemble them frequently to lubricate those parts. In this regard, for the sake of reliable and accurate manipulations of the robotic device in practice, the friction is worth being considered in the inverse dynamic model to analyse its effects.

In terms of friction effects in PMs, from the literature, a Lagrange-based approach was proposed to analyse the forward dynamics of a 3-PRS PM in [24], where the LuGre friction effects are modelled at active prismatic joints. Letters P and S denote a prismatic joint and a spherical joint, respectively. On this basis, the influence on the motion accuracy was analysed. EOMs of forward dynamics with a closed form were formatted by the Udwadia–Kalaba approach for a planar 2RRR/RR PM with actuation redundancy in [31]. In [32], the friction effects were expressed by the Stribeck model in the artificial hip joint, thrust ball bearing, and linear module of 2(3PUS+S) PM. The letter U denotes a universal joint. Their parameters were identified by a least-squares method, and their negative effects were compensated for through a feedforward compensation method. An explicit dynamic model with joint friction was established for a 4-UPS PM, which was incorporated into a 5-DOF hybrid polishing robot [14]. The Newton–Euler method was utilised to build the model, and friction effects were modelled by the Coulomb-viscous friction model. To build an accurate dynamic model of a hybrid spray-painting robot in [33], the Stribeck friction model was used to express friction effects in all active joints. The coefficients were identified, and these effects were compensated for in the inverse dynamic model. A nonlinear friction model, which can capture viscous, Coulomb, and Stribeck effects, was developed for the 2RRR/RR PM in [34]. The comparison of the control performance between this model and the Coulomb-viscous friction model showed that the trajectory tracking accuracy was improved significantly in the former.

From the literature review, the practical applications of Euler parameters can be found extensively, and their theoretical study has reached an in-depth level. However, their application in PMs, especially those with various constraints from lower kinematic pairs and HKPs like the one under study, has not been reported yet. In terms of friction effects, they are considered only in a few publications about the dynamics of PMs. In the target PM, to reduce the computational demands of inverse dynamics and the negative effects from friction, an Euler parameter-based inverse dynamic model with friction effects at HKPs and actuators is built in this paper. The two novelties are summarised as:

1. Euler parameters are employed to describe the motions of the constrained end effector in the target PM, sharply reducing the computational expenditure of the model.
2. Friction effects are accounted for at HKPs and active revolute joints, arriving at a nonlinear but more accurate inverse dynamic model.

The paper is organised as follows: in Section 2, the mechanism under study is described in detail. The motions of the end effector are studied using Euler parameters in Section 3. In Section 4, the inverse dynamic model with friction effects is built by Newton–Euler’s law. Numerical computations are presented in Section 5 to find the transformation between Euler parameters and Euler angles, the dynamic performances of the mechanism under study, and the influence of constraints at HKPs on kinematics and dynamics. Finally, some conclusions are given in Section 6.

## 2. The Robotic Mechanism

**Figure 1:**
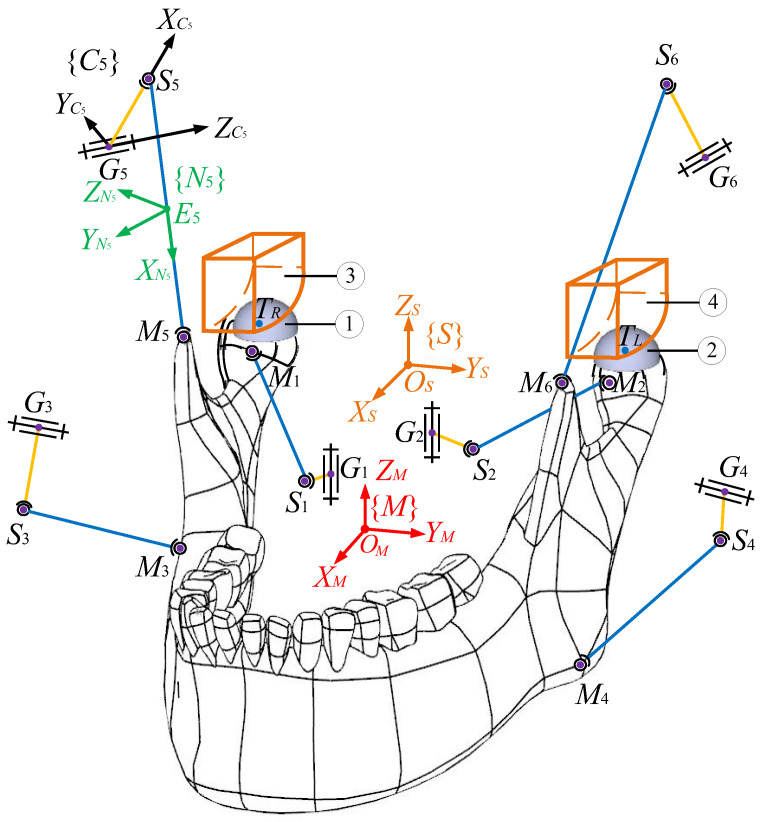
A schematic view of the mechanism under study: ① and ② are condylar balls, and ③ and ④ are articular surfaces of TMJs.

The scheme of the target PM constrained at two HKPs is illustrated in Figure 1. The fixed maxilla to which the inertia frame {*S*} is assigned is not shown in the figure for a clear exhibition of moving bodies. The movable lower jaw, namely, the end effector, is connected to the base by six independent kinematic linkages. Frame {*S*} owns a horizontal *X_S_*-*Y_S_* plane perpendicular to the vertical *Z_S_* axis. At the mass centre *O_M_* of the end effector, a body-fixed frame {*M*} is set. Frames {*S*} and {*M*} are completely coincident when the mechanism is at the home position, i.e., the upper and lower jaws are occlusal. The position coordinates of *O_M_* in {*S*} denote the translations of the end effector, and its orientations with respect to the inertia frame are computed by Euler parameters. In the chains, the crank ***G****_i_**S**_i_* (*i* = 1,…,6) is actuated by a revolute joint centred at *G_i_*, and the coupler ***S****_i_**M**_i_* connects the crank and the end effector at its two ends *S_i_* and *M_i_*, respectively, using spherical joints. A frame {*C_i_*} is attached to *G_i_* to express the rotation of the *i*th actuator. In it, the $X_{C_{i}}$ axis is from *G_i_* to *S_i_*, the $Z_{C_{i}}$ axis runs through the rotational axis of the actuator, and the $Y_{C_{i}}$ axis completes the frame, obeying the right-hand rule. A frame {*N_i_*} is attached to the mass centre *E_i_* of the coupler to describe its motions in {*S*}. The $X_{N_{i}}$ axis points from *S_i_* to *M_i_*, the $Y_{N_{i}}$ axis is parallel to the cross product of two unit vectors defined along the $X_{N_{i}}$ and *X_S_* axes, and the $Z_{N_{i}}$ axis is defined by the right-hand rule. Note that only one actuator frame {*C*_5_} and one coupler frame {*N*_5_} are drawn as illustrative examples in the diagram for the sake of clarity.

**Figure 2:**
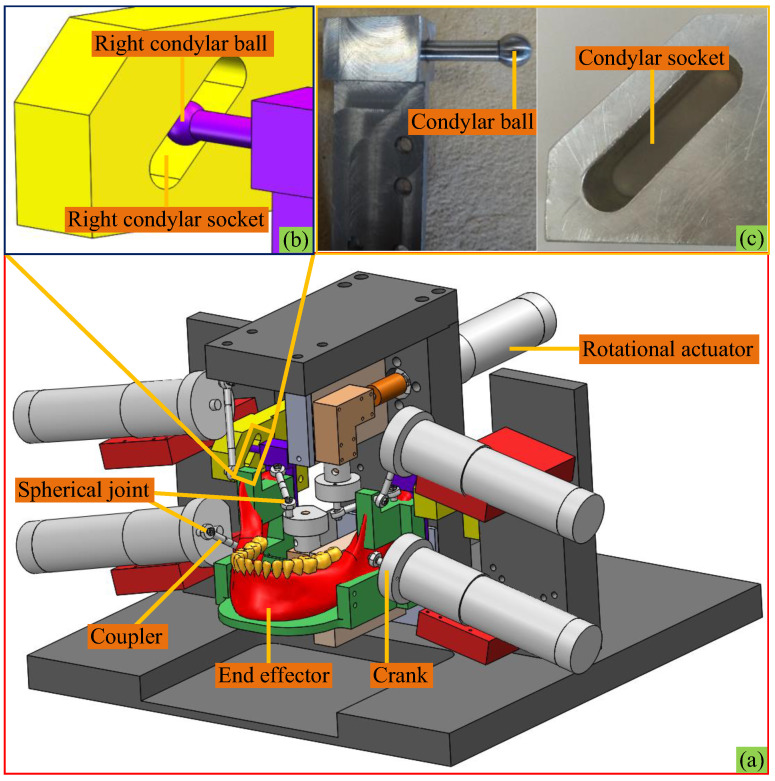
The mechanism under study: (**a**) CAD model, (**b**) HKP at the right side, (**c**) HKP-related mechanical parts.

From a close observation of Figure 1, one can see that the point-contact HKPs mimicking TMJs between left and right condylar balls and condylar surfaces are only schematic, since the former can only receive unilateral constraints from the latter. Hence, in the prototype, due to motion errors in engineering practice, condylar balls are separated from articular surfaces easily. Thus, the nature of the mechanism is changed. In these regards, the HKP-related mechanical parts in engineering practice are shown in Figure 2 with the computer-aided design (CAD) model of the prototype. The condylar ball slips along a condylar socket that has a width equal to its diameter. Thereby, the point-contact HKPs during the arbitrary motions of the end effector can always exist. By this design, the motion of the condyle ball centre *T_i_* (*i* = *L*, *R*) is always constrained onto a surface, which is offset from the upper and lower surfaces of the socket by the ball radius. Thereupon, it is clear that the end effector is actuated by six chains and constrained by the base at two HKPs simultaneously.

## 3. Kinematics

The kinematics of the end effector using Euler parameters is analysed in this section so as to be utilised adequately in the dynamics in Section 4.

### 3.1. Constrained Motions of the End Effector

The influence of DCFB on the end effector is first derived. In the inertia frame {*S*}, the mathematical functions of the planar surfaces where the condylar ball centres *T_i_* (*i* = *L*, *R*) are situated

(2) $$p_{1}\cdot X_{S}+Z_{S}+p_{2}=0,\;\;p_{3}\leq X_{S}\leq p_{4},\;\;p_{5}\leq Y_{S}^{L}\leq p_{6},\;\;-p_{6}\leq Y_{S}^{R}\leq -p_{5}$$

where $Y_{S}^{i}\left(i=L,R\right)$ are the coordinates of the left and right planes along the *Y_S_* axis, respectively.

The coordinates of *T_i_* (*i* = *L*, *R*) in {*S*} are

(3) OSTi=OSOM+RMSθ⋅OMMTi

where $O_{S}O_{M}={\left[\begin{array}{ccc} X & Y & Z \end{array}\right]}^{T}$ denotes the position coordinates of the mass centre *O_M_* in {*S*}, OMMTi is the position vector of ***T****_i_* in {*M*}, which is constant, and RMSθ rotates frame {*S*} to frame {*M*} using Euler parameters ***θ*** and is expressed in a quadratic form

(4) RMSθ=2⋅θ02−1⋅I3+2θ⋅θT+θ0⋅θ×

where $\theta =\left[\begin{array}{c} \theta_{0}\\ \theta \end{array}\right]$,$\theta =\left[\begin{array}{c} \theta_{1}\\ \theta_{2}\\ \theta_{3} \end{array}\right]$, ***I***_3_ is the 3 × 3 identity matrix, and θ× is the 3 × 3 cross-product matrix spanned by θ. Critically, θ fulfils the normality constraint condition

(5) $$\theta^{T}\cdot \theta =1.$$

Therefore, the four quantities in θ are not all independent.

Substituting Equations (3) and (4) into Equation (2) produces the geometric constraint equation at two HKPs as

(6) p1⋅X+R1,:MS⋅OMMTj+Z+R3,:MS⋅OMMTj+p2=0,j=L,R

where Ri,:MS is the *i*th (*i* = 1,3) row of RMS. Owing to the left-right symmetry of OMMTL and OMMTR in {*M*}, a summation and a subtraction of the two equations in Equation (6) sidewise yield

(7) Z=−p1X+p2+p1⋅R1,:MS+R3,:MS⋅OMMTL10OMMTL3θ3=p1⋅θ2+θ0p1⋅θ0−θ2⋅θ1

where OMMTLii=1,3 denotes the *i*th element of OMMTL. Thus, under DCFB, *Z* and $\theta_{3}$ are parasitic motion variables, rather than DOFs of the 6RSS PM free of these constraints. Specifically, *Z* is constituted by both a linear DOF *X* and Euler parameters θ contained in Ri,:MSi=1,3, while $\theta_{3}$ is only composed of the other three elements in θ. Hence, the four entries in θ are further coupled on the basis of Equation (5).

Equation (7) has purely algebraic functions but no trigonometric functions. By comparison, the parasitic motion variable *Z* in Equation (6) of [20] has the same expression as that in Equation (7), but the rotation matrix RMS in it is full of trigonometric functions.

The seven coordinates describing the motion of the end effector are grouped as

(8) $$X_{EE}^{P}={\left[\begin{array}{cccc} X & Y & Z & \theta^{T} \end{array}\right]}^{T}$$

In view of the three scalar equations in Equations (5) and (7), the mechanism has four DOFs, which is also in accordance with the result from the Kutzbach–Grübler criterion [35]. Remember the six actuations are invariant; thus, redundant actuations in the mechanism are essentially caused by DCFB onto the end effector, which is completely different from the two primary accesses in [36].

After identifying the parasitic motions, five independent coordinates of $X_{EE}^{P}$ are used to express the four DOFs and grouped in a 5 × 1 vector $q_{EE}^{P}$ as

(9) $$q_{EE}^{P}={\left[\begin{array}{ccccc} X & Y & \theta_{0} & \theta_{1} & \theta_{2} \end{array}\right]}^{T}$$

Note that it is not four coordinates of $X_{EE}^{P}$ that are used to express the DOFs of the mechanism. Two reasons account for this choice. Firstly, putting *θ*_3_ in Equation (7) into the quadratic constraint in Equation (5) can produce a complicated expression of *θ_i_* (*i* = 0,1,2), which is not in favour of the computational cost. Secondly, via the optimisation in Section 5.1, all variables in $X_{EE}^{P}$ can be conveniently obtained numerically.

Henceforth, to characterise an instantaneous configuration of the end effector, Equation (8), or Equations (5), (7), and (9) ad hoc are needed. In other words, the mechanism performs motions in six directions with five independent coordinates, two parasitic motion variables, and one normality constraint condition.

The 7 × 5 Jacobian matrix ***M****_J_* between ${\dot{q}}_{EE}^{P}$ and ${\dot{X}}_{EE}^{P}$ as

(10) $${\dot{X}}_{EE}^{P}=M_{J}\cdot {\dot{q}}_{EE}^{P}$$

Differentiating this equation produces

(11) $${\ddot{X}}_{EE}^{P}=\left[\begin{array}{cc} {\dot{M}}_{J} & M_{J} \end{array}\right]\cdot \left[\begin{array}{c} {\dot{q}}_{EE}^{P}\\ {\ddot{q}}_{EE}^{P} \end{array}\right]$$

The twist of the end effector can be computed as

(12) $$t_{EE}=\left[\begin{array}{c} V_{O_{M}}\\ \omega_{EE}^{P} \end{array}\right]=M_{0b}\cdot {\dot{X}}_{EE}^{P}=M_{1b}\cdot {\dot{q}}_{EE}^{P}$$

where $V_{O_{M}}$ is the translational velocity of *O_M_*, $\omega_{EE}^{P}$ is the rotational velocity, and

(13) $$\begin{array}{l} M_{0b}=diag\left(I_{3}\;2\cdot E_{EE}\right)\\ E_{EE}=\left[-\theta ,\;\theta_{0}\cdot I_{3}+\theta \times \right]\\ M_{1b}=M_{0b}\cdot M_{J} \end{array}$$

On this basis, the first time-rate of the twist is easily derived as

(14) $${\dot{t}}_{EE}=\left[\begin{array}{c} {\dot{V}}_{O_{M}}\\ {\dot{\omega }}_{EE}^{P} \end{array}\right]=\left[\begin{array}{cc} {\dot{M}}_{1b} & M_{1b} \end{array}\right]\cdot \left[\begin{array}{c} {\dot{q}}_{EE}^{P}\\ {\ddot{q}}_{EE}^{P} \end{array}\right]$$

It is noted that though there are some common figures, equations, and texts between this paper and [20], they are in Section 2 and Section 3.1, serving as the foundation of this paper, since the focus of this paper is on the use of Euler parameters and the friction effects, and these two aspects do not exist in [20]. Finally, the dynamic models in this manuscript and [20] are built by Newton–Euler’s law and the energy-based methods, respectively. Therefore, these replications are not critical.

### 3.2. Crank G_i_S_i_

The crank only rotates around the fixed $Z_{C_{i}}$ axis of frame {*C_i_*}; however, it is not easy to express its rotations using Euler parameters $\theta_{G_{i}S_{i}}=\left[\begin{array}{c} \theta_{0G_{i}S_{i}}\\ \theta_{G_{i}S_{i}} \end{array}\right]$ where $\theta_{G_{i}S_{i}}={\left[\begin{array}{ccc} \theta_{1G_{i}S_{i}} & \theta_{2G_{i}S_{i}} & \theta_{3G_{i}S_{i}} \end{array}\right]}^{T}$. The reason is as follows: from the geometric relationship, one can derive

(15) GiSi=RCi0S⋅RCiCi0θGiSi⋅GiCiSi

in which RCi0S is the rotation matrix of ***G****_i_****S****_i_* in {*S*} at the initial configuration of the mechanism, RCiCi0θGiSi is the rotation matrix about the $Z_{C_{i}}$ axis

(16) RCiCi0θGiSi=2⋅θ0GiSi2−1⋅I3+2θGiSi⋅θGiSiT+θ0GiSi⋅θGiSi×

and GiCiSi=GiSi02×1 where $\left\|G_{i}S_{i}\right\|$ is the length of $G_{i}S_{i}$. On the one hand, as depicted in Figure 1, one can find

(17) $$S_{i}M_{i}=G_{i}M_{i}-G_{i}S_{i}\;$$

then squaring the two sides of Equation (17) and rewriting the results produce

(18) $$G_{i}M_{i}^{T}\cdot G_{i}S_{i}=\frac{{\left\|S_{i}M_{i}\right\|}^{2}-{\left\|G_{i}M_{i}\right\|}^{2}-{\left\|G_{i}S_{i}\right\|}^{2}}{-2}$$

On the other hand, one can find

(19) $$G_{i}M_{i}=G_{i}O_{S}+O_{S}O_{M}+O_{M}M_{i}$$

Since the crank ***G****_i_****S****_i_*  can only rotate around the $Z_{C_{i}}$ axis, the four elements in $\theta_{G_{i}S_{i}}$ are

(20) $$\begin{array}{l} \theta_{0G_{i}S_{i}}=\cos\frac{\theta_{G_{i}S_{i}}}{2}\\ \theta_{1G_{i}S_{i}}=\theta_{2G_{i}S_{i}}=0\\ \theta_{3G_{i}S_{i}}=\sin\frac{\theta_{G_{i}S_{i}}}{2} \end{array}$$

where $\theta_{G_{i}S_{i}}$ cis the angular displacement of the *i*th active revolute joint around the $Z_{C_{i}}$ axis. In this manner, putting Equations (15), (16), and (19), $\theta_{1G_{i}S_{i}}$ and $\theta_{2G_{i}S_{i}}$ in Equation (20) into Equation (18) gives rise to a scalar equation

(21) GiMiT⋅RCi0S⋅GiSi⋅2⋅θ0GiSi2−12⋅θ0GiSi⋅θ3GiSi0=RHSGiSi

where $RHS_{G_{i}S_{i}}$ is the scalar on the right-hand side of Equation (18). However, it can be shown from Equation (21) that it is hard to compute $\theta_{0G_{i}S_{i}}$ and $\theta_{3G_{i}S_{i}}$ simultaneously, since in general, nine numerical values of a 3 × 3 rotation matrix are all needed, as drawn from the conclusion after reviewing the various sorts of methods in [37]. However, if $\theta_{0G_{i}S_{i}}$ and $\theta_{3G_{i}S_{i}}$ in Equation (20) are substituted into (21), $\theta_{G_{i}S_{i}}$ can be derived using the procedure in Section 2 of [2]. In fact, substituting Equation (20) into Equation (16) easily produces

(22) RCiCi0=cosθGiSi−sinθGiSi0sinθGiSicosθGiSi0001

which is actually the rotation matrix by the Euler angle $\theta_{G_{i}S_{i}}$ around the $Z_{C_{i}}$ axis. In these regards, the kinematics of the *i*th actuator including its displacement, velocity, and acceleration is still derived as that in Section 2 of [2] and Section 3.2 of [20] using $\theta_{G_{i}S_{i}}$.

### 3.3. Coupler S_i_M_i_

The motions of the coupler ***S****_i_**M**_i_* (*i* = 1,…,6) are also needed for its rigid-body dynamics. On the one hand, the coordinate vector of ***S****_i_**M**_i_* is computed as

(23) SiMi=RNi0S⋅RNiNi0θSiMi⋅SiMi02×1

where RNi0S is the rotation matrix of ***S****_i_**M**_i_* in {*S*} at the initial configuration of the mechanism; $\left\|S_{i}M_{i}\right\|$ is the length of ***S****_i_**M**_i_*, and RNiNi0θSiMi is the rotation matrix since the configuration using Euler parameters $\theta_{S_{i}M_{i}}$

(24) RNiNi0θSiMi=2θ0SiMi−1⋅I3+2θSiMi⋅θSiMiT+θ0SiMi⋅θSiMi×

where

$$\begin{array}{l} \theta_{S_{i}M_{i}}={\left[\begin{array}{cc} \theta_{0S_{i}M_{i}} & \theta_{S_{i}M_{i}}^{T} \end{array}\right]}^{T}\\ \theta_{S_{i}M_{i}}={\left[\begin{array}{ccc} \theta_{1S_{i}M_{i}} & \theta_{2S_{i}M_{i}} & \theta_{3S_{i}M_{i}} \end{array}\right]}^{T} \end{array}$$

On the other hand, geometrically, ***S****_i_**M**_i_* can also be computed from the differences of the coordinate vector of ***M****_i_* and ***S****_i_* as

(25) $$S_{i}M_{i}=O_{S}O_{M}+O_{M}M_{i}-O_{S}G_{i}-G_{i}S_{i}$$

where OMMi=RMSθ⋅OMMMi and OMMMi is the constant coordinate vector of *M_i_* in {*M*}, $O_{S}G_{i}$ is the constant position vector of *G_i_* since it is fixed in {*S*}, and $G_{i}S_{i}$ is derived using Equations (15) and (22). When computing $\theta_{S_{i}M_{i}}$, numerical values of the right-hand side of Equation (25) are all available, i.e., the motions of the end effector and the crank $G_{i}S_{i}$ have been derived before. Combining Equations (23)–(25), one can find

(26) 2θ0SiMi2+θ1SiMi2−12θ1SiMi⋅θ2SiMi+θ0SiMi⋅θ3SiMi2θ1SiMi⋅θ3SiMi−θ0SiMi⋅θ2SiMi=R−1Ni0SSiMi⋅RHSSiMi

whose left-hand side is actually the first column of RNiNi0θSiMi from Equation (24), and $RHS_{S_{i}M_{i}}$ is the known 3 × 1 vector at the right-hand side of Equation (25). By taking the normality constraint equation

(27) $$\theta_{0S_{i}M_{i}}^{2}+\theta_{1S_{i}M_{i}}^{2}+\theta_{2S_{i}M_{i}}^{2}+\theta_{3S_{i}M_{i}}^{2}=1$$

into consideration, there are in total four unknowns and four nonlinear scalar equations which can be solved numerically, being neither simple nor straightforward. However, the mechanism is featured by the end effector constrained by the base directly. As such, the rotations of the couplers by virtue of Euler parameters will be left for future work. In summary, in the kinematics of the mechanism, Euler parameters are only utilised in the end effector, while Euler angles in the kinematic chains, as in [2] and [20], are still employed to describe their rotations.

## 4. Dynamic Model

Before the dynamic model is established, two reasonable assumptions about friction effects are made: 1. The two HKPs are easily subject to wear and tear due to friction forces. Thereby, from the viewpoint of practical applications, these forces are incorporated into the dynamic model to make it more accurate. Meanwhile, in the prototype, revolute joints are actuated by DC servo motors, and it is hard to lubricate the inner bodies, such as brushes, rotors, or bearings. Henceforth, friction torques in these joints are also modelled. 2. Frictional moments exist in twelve passive spherical joints; however, the size of the ball in the socket is very small, so these moments are not large. Additionally, friction effects in passive spherical joints of PMs have been studied in [14] already. To avoid a tedious deviation in the dynamic model and highlight the unique HKPs, all spherical joints are assumed friction-free.

Friction effects are tightly related to constraint forces at joints, which are difficult to find using energy-based dynamic methods, such as the Lagrangian formulation or Hamilton’s equations. Additionally, in view of the intrinsic dependence of Euler parameters, Lagrange multipliers are needed if Lagrange equations are used to build the model. They would greatly increase the number of coordinates in the model. Consequently, the methods in [21,38] are not adopted, and the dynamic model of the entire mechanism is built using the classical Newton–Euler’s law. A great deal of friction models have been reviewed in [39], which are classified into static and dynamic types. In view of the complex topology of the target mechanism, the classical Coulomb and viscous friction model, which belongs to the static type, is applied.

### 4.1. End Effector

**Figure 3:**
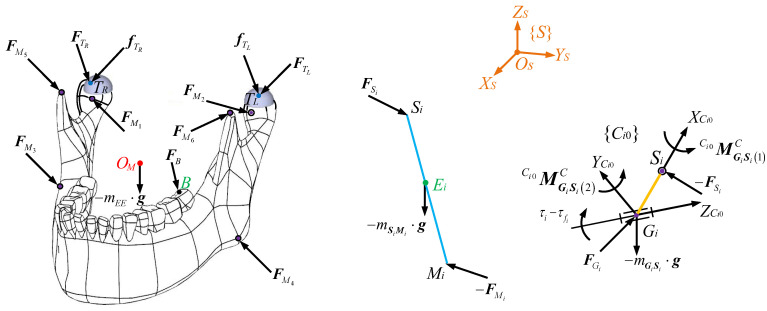
Free-body diagram of the end effector, the *i*th (*i* = 1,…,6) coupler, and the *i*th crank.

**Figure 4:**
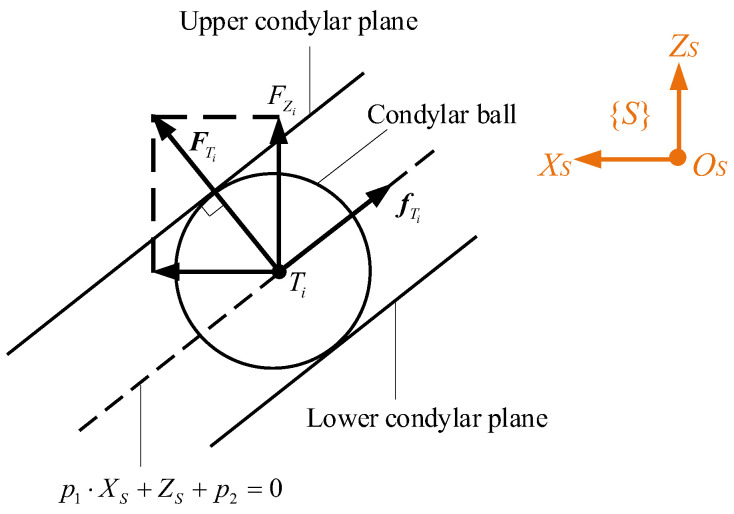
Schematic diagram of the constraint forces $F_{T_{i}}$ from condylar sockets and the friction forces $f_{T_{i}}$ at the condylar ball.

The free-body diagram of the end effector is shown in Figure 3. The forces acting on the end effector include constraint forces $F_{M_{i}}\left(i=1,\ldots ,6\right)$ at spherical joints *M_i_*, its gravity $-m_{EE}\cdot g$ at *O_M_* in which *m_EE_* is the mass and $g={\left[\begin{array}{cc} 0_{2\times 1} & 9800 \end{array}\right]}^{T}mm/s^{2}$ is the gravitational acceleration vector, the reacted chewing force ***F****_B_* at point *B* of a left lower molar, and constraint forces $F_{T_{i}}\left(i=L,R\right)$ and friction forces $f_{T_{i}}$ acting at *T_i_* from the surfaces of condylar sockets. $F_{T_{i}}$ can be computed as

(28) $$F_{T_{i}}=M_{H}\cdot F_{Z_{i}}$$

where $M_{H}={\left[\begin{array}{ccc} 1.1 & 0 & 1 \end{array}\right]}^{T}$ is along the orthogonal direction of the planar surface specified in Equation (2), and $F_{Z_{i}}$ is the component of $F_{T_{i}}$ along the *Z_S_* axis. The friction force at HKPs under the Coulomb and viscous friction model is

(29) $$f_{T_{i}}=\left\{\begin{array}{c} \left(A_{i}\cdot \left|F_{Z_{i}}\right|+\mu_{V}\right)\cdot V_{T_{i}},\;\;\;V_{T_{i}}\neq 0_{3\times 1},\;\;\;A_{i}=\mu_{C}\frac{\sqrt{p_{1}^{2}+1}}{\left\|V_{T_{i}}\right\|}\\ 0_{3\times 1},\;\;\;V_{T_{i}}=0_{3\times 1} \end{array}\right.$$

where $\mu_{C}$ and $\mu_{V}$ are Coulomb and viscous coefficients, respectively, $\left|F_{Z_{i}}\right|$ is the absolute value of $F_{Z_{i}}$, and $\left\|V_{T_{i}}\right\|$ is the two-norm sum of the linear velocity $V_{T_{i}}$ at *T_i_*. As a result, friction forces at HKPs are not only a function of $V_{T_{i}}$ but also the dynamics of the entire mechanism. The schematic diagram of the constraint forces $F_{T_{i}}$ from condylar sockets and the friction forces $f_{T_{i}}$ at the condylar ball from the sagittal view is displayed in Figure 4. One can see that $F_{T_{i}}$ is perpendicular to the surface on which *T_i_* is constrained, and $f_{T_{i}}$ is on this planar surface. $F_{T_{i}}$ can be decomposed into two components, which are along the *Z_S_* and *X_S_* axes, respectively.

Note that since at all time instants of the tracked trajectory in Section 5, $V_{T_{i}}$ is nonzero, then the case $f_{T_{i}}=0_{3\times 1}$ is not considered in the following. By the Newton–Euler formulation, the EOMs of the lower jaw are

(30) $$M_{2b}\cdot F_{M_{1\_6}}+{\bar{M}}_{3b}\cdot \left[\begin{array}{c} F_{T_{L}}-f_{T_{L}}\\ F_{T_{R}}-f_{T_{R}} \end{array}\right]={\bar{M}}_{4b}$$

where

(31) $$\begin{array}{l} M_{2b}=\left[\begin{array}{ccc} I_{3} & \ldots & I_{3}\\ O_{M}M_{1}\times & \ldots & O_{M}M_{6}\times \end{array}\right],\;{\bar{M}}_{3b}=\left[\begin{array}{cc} I_{3} & I_{3}\\ O_{M}T_{L}\times & O_{M}T_{R}\times \end{array}\right]\\ F_{M_{1\_6}}=\left[\begin{array}{c} F_{M_{1}}\\ \vdots \\ F_{M_{6}} \end{array}\right],\;{\bar{M}}_{4b}=\left[\begin{array}{c} m_{EE}\left({\dot{V}}_{O_{M}}+g\right)\\ I_{EE}\cdot {\dot{\omega }}_{EE}+\omega_{EE}\times \left(I_{EE}\cdot \omega_{EE}\right) \end{array}\right]-W_{F_{B}} \end{array}$$

In Equation (31), $O_{M}M_{i}\times \left(i=1,\ldots ,6\right)$ and $O_{M}T_{i}\times \left(i=L,R\right)$ are cross-product matrices spanned by $O_{M}M_{i}$ and $O_{M}T_{i}$, respectively, $W_{F_{B}}$ is the reacted wrench on the end effector by ***F****_B_*. *m_EE_* is the mass of the end effector, IEE=RMS⋅IEEM⋅RTMS is the inertia matrix of the end effector updated as a function of its orientations in {*S*}, and IEEM is the inertia tensor of the end effector with respect to {*M*}.

Putting Equations (28) and (29) into Equation (30) gives rise to a compact form as

(32) $$M_{2b}\cdot F_{M_{1\_6}}+M_{3b}\cdot F_{Z}-{\bar{M}}_{5b}\cdot \left|F_{Z}\right|=M_{4b}$$

where

$$\begin{array}{l} M_{3b}={\bar{M}}_{3b}\cdot \left(I_{2}⊗M_{H}\right),\;{\bar{M}}_{5b}={\bar{M}}_{3b}\cdot diag\left(\begin{array}{cc} A_{L}\cdot V_{T_{L}} & A_{R}\cdot V_{T_{R}} \end{array}\right)\\ F_{Z}=\left[\begin{array}{c} F_{Z_{L}}\\ F_{Z_{R}} \end{array}\right],\;\;\;\;\;M_{4b}={\bar{M}}_{4b}+{\bar{M}}_{3b}\cdot \mu_{V}\cdot \left[\begin{array}{c} V_{T_{L}}\\ V_{T_{R}} \end{array}\right] \end{array}$$

and ⊗ is the Kronecker product.

### 4.2. Coupler S_i_M_i_

The free-body diagram of the *i*th $\left(i=1,\ldots ,6\right)$ coupler is given in Figure 3. Via the Newton–Euler’s law, for the *i*th coupler, one can format

(33) $$\begin{array}{l} F_{S_{i}}-F_{M_{i}}=m_{S_{i}M_{i}}\cdot \left({\dot{V}}_{E_{i}}+g\right)\\ E_{i}S_{i}\times F_{S_{i}}+E_{i}M_{i}\times \left(-F_{M_{i}}\right)=I_{S_{i}M_{i}}\cdot {\dot{\omega }}_{S_{i}M_{i}}+\omega_{S_{i}M_{i}}\times \left(I_{S_{i}M_{i}}\cdot \omega_{S_{i}M_{i}}\right)={\bar{E}}_{S_{i}M_{i}} \end{array}$$

where $F_{S_{i}}$ is the constraint forces at *S_i_* acting at ***S****_i_**M**_i_*, $m_{S_{i}M_{i}}$ is the mass, and ${\dot{V}}_{E_{i}}$ is the linear acceleration of *E_i_*, $\omega_{S_{i}M_{i}}$ and ${\dot{\omega }}_{S_{i}M_{i}}$ are the angular velocity and acceleration, respectively, ISiMi=RNiS⋅ISiMiNi⋅RTNiS is the inertia tensor with respect to *E_i_* and built in {*S*}, and ISiMiNi is the inertia tensor with respect to *E_i_* and built in {*N_i_*}. Combining the two equations in Equation (33) yields

(34) $$M_{i}S_{i}\times F_{M_{i}}=E_{S_{i}M_{i}}$$

where $E_{S_{i}M_{i}}={\bar{E}}_{S_{i}M_{i}}+0.5\cdot m_{S_{i}M_{i}}\cdot S_{i}M_{i}\times \left({\dot{V}}_{E_{i}}+g\right)$.

Among the three scalar equations in Equation (34), arbitrarily only two are independent; the last two are chosen for the following computation. Thus, for the six couplers, one can write

(35) $$M_{5b}\cdot F_{M_{1\_6}}=M_{6b}$$

where $M_{5b}=diag\left({\left(M_{1}S_{1}\times \right)}_{\left(2:3,:\right)}\;\ldots \;{\left(M_{6}S_{6}\times \right)}_{\left(2:3,:\right)}\right),M_{6b}=\left[\begin{array}{c} E_{S_{1}M_{1}\left(2:3\right)}\\ \vdots \\ E_{S_{6}M_{6}\left(2:3\right)} \end{array}\right]$, and the subscripts (2:3,:) and (2:3) denote the last two rows of $M_{i}S_{i}\times$ and the last two entries of $E_{S_{i}M_{i}}$, respectively. Meanwhile, repeating the first equation in Equation (33) six times for the six couplers produces

(36) $$F_{S_{1\_6}}=F_{M_{1\_6}}+M_{7b}$$

where

$$F_{S_{1\_6}}=\left[\begin{array}{c} F_{S_{1}}\\ \vdots \\ F_{S_{6}} \end{array}\right],\;M_{7b}=\left[\begin{array}{c} m_{S_{1}M_{1}}\cdot \left({\dot{V}}_{E_{1}}+g\right)\\ \vdots \\ m_{S_{6}M_{6}}\cdot \left({\dot{V}}_{E_{6}}+g\right) \end{array}\right]$$

These equations will be incorporated into those for the end effector and crank to build the dynamic model of the entire mechanism.

### 4.3. Crank G_i_S_i_

To find frictional torques at active revolute joints at *G_i_*, the constraint force $F_{G_{i}}$ is to be derived. The free-body diagram of the *i*th $\left(i=1,\ldots ,6\right)$ crank is shown in Figure 3. Firstly, because the crank owns a cylinder shape, and it only rotates around the central line, which is along the $Z_{C_{i}}$ axis of frame {*C_i_*}, its force equilibrium in {*S*} produces

(37) $$F_{G_{i}}=F_{S_{i}}+m_{G_{i}S_{i}}\cdot g$$

where $m_{G_{i}S_{i}}$ is the mass and its mass centre locates at the $Z_{C_{i}}$ axis. $F_{G_{i}}$ can be expressed in frame {*C_i_*_0_} as

(38) FGiCi0=RSCi0⋅FGi=RTCi0S⋅FSi+mGiSi⋅g

to minimise computational overhead, where frame {*C_i_*_0_} denotes frame {*C_i_*} when the angular displacement $\theta_{G_{i}S_{i}}$ of the *i*th crank is zero. As such, for the six revolute joints, a compact form of all constraint forces at *G_i_* can be written as

(39) FG1C10⋮FG6C60=diagRTC10S…RTC60S⋅FS1_6+mG1S1⋅g⋮mG6S6⋅g

The Euler’s law is used to write the rotational EOM of the *i*th crank in {*C_i_*_0_} as

(40) GiCi0Si×FSiCi0+MGiSiCCi0τi−τfi=02×1IGiSi⋅θ¨GiSi

where MGiSiCCi0 is the 2 × 1 vector containing two constraint moments around the $X_{C_{i}}$ and $Y_{C_{i}}$ axes of {*C_i_*_0_}, $\tau_{i}$ and $\tau_{f_{i}}$ are the actuating torque and the frictional torque in the *i*th actuator, respectively, ${\ddot{\theta }}_{G_{i}S_{i}}$ is the angular acceleration, and $I_{G_{i}S_{i}}$ is the rotational inertia of the *i*th crank. Thereby, around the direction of actuations, i.e., from the third line of Equation (40), one can list

(41) IGiSi⋅θ¨GiSi=τi−τfi+GiCi0Si×3,:⋅FSiCi0

where GiCi0Si× is the cross-product matrix of GiCi0Si and GiCi0Si×3,: means the third line. By the Coulomb and viscous friction model, $\tau_{f_{i}}$ is computed as

(42) τfi=Ri⋅μCi⋅sgnθ˙GiSi⋅FGi1:2Ci0+μVi⋅θ˙GiSi

where *R_i_* is the friction arm of the *i*th actuator, $\mu_{C_{i}}$ and $\mu_{V_{i}}$ are the Coulomb and viscous coefficients, respectively, and sgn(·) is the sign function. Additionally, FSiCi0 can be written as

(43) FSiCi0=RSCi0⋅−FSi=RTCi0S⋅−FSi

Putting it into Equation (40) produces

(44) $$\tau_{i}=\tau_{f_{i}}+I_{G_{i}S_{i}}\cdot {\ddot{\theta }}_{G_{i}S_{i}}+M_{8bi}\cdot F_{S_{i}}$$

where M8bi=GiCi0Si×3,:⋅RTCi0S. As a result, for the six cranks, one can derive

(45) $$\tau =\tau_{f}+M_{9b}+M_{8b}\cdot F_{S_{1\_6}}$$

where

$$\tau =\left[\begin{array}{c} \tau_{1}\\ \vdots \\ \tau_{6} \end{array}\right],\;\tau_{f}=\left[\begin{array}{c} \tau_{f_{1}}\\ \vdots \\ \tau_{f_{6}} \end{array}\right],\;M_{8b}=diag\left(\begin{array}{ccc} M_{8b1} & \ldots & M_{8b6} \end{array}\right),\;M_{9b}=\left[\begin{array}{c} I_{G_{1}S_{1}}\cdot {\ddot{\theta }}_{G_{1}S_{1}}\\ \vdots \\ I_{G_{6}S_{6}}\cdot {\ddot{\theta }}_{G_{6}S_{6}} \end{array}\right]$$

In addition, from the first two lines of Equation (40), one can write

(46) MGiSiCCi0=−GiCi0Si×1:2,:⋅FSiCi0

where GiCi0Si×1:2,: is the first two lines of GiCi0Si×.

### 4.4. Entire Mechanism

Combining the EOMs of the end effector in Equation (32) and the six couplers in Equation (35) generates

(47) $$\left[\begin{array}{c} M_{2b}\\ M_{5b} \end{array}\right]\cdot F_{M_{1\_6}}=\left[\begin{array}{c} M_{3b}\\ M_{6b} \end{array}\right]-\left[\begin{array}{c} M_{4b}\\ 0_{12\times 2} \end{array}\right]\cdot F_{Z}+\left[\begin{array}{c} {\bar{M}}_{5b}\\ 0_{12\times 2} \end{array}\right]\cdot \left|F_{Z}\right|$$

Then, $F_{M_{1\_6}}$ can be computed as

(48) $$F_{M_{1\_6}}=M_{10b}-M_{11b}\cdot F_{Z}+M_{12b}\cdot \left|F_{Z}\right|$$

where

$$M_{10b}={\left[\begin{array}{c} M_{2b}\\ M_{5b} \end{array}\right]}^{-1}\cdot \left[\begin{array}{c} M_{3b}\\ M_{6b} \end{array}\right],\;M_{11b}={\left[\begin{array}{c} M_{2b}\\ M_{5b} \end{array}\right]}^{-1}\cdot \left[\begin{array}{c} M_{4b}\\ 0_{12\times 2} \end{array}\right],\;M_{12b}={\left[\begin{array}{c} M_{2b}\\ M_{5b} \end{array}\right]}^{-1}\cdot \left[\begin{array}{c} {\bar{M}}_{5b}\\ 0_{12\times 2} \end{array}\right]$$

Thus, with Equation (36), all constraint forces at *S*_1_~*S*_6_ can be computed as

(49) $$F_{S_{1\_6}}=M_{13b}-M_{11b}\cdot F_{Z}+M_{12b}\cdot \left|F_{Z}\right|$$

where $M_{13b}=M_{10b}+M_{7b}$. Finally, putting Equation (49) into Equation (45) produces the explicit dynamic model of the entire mechanism with friction effects at HKPs and revolute joints as

(50) $$\tau =\tau_{f}+M_{14b}-M_{15b}\cdot F_{Z}+M_{16b}\cdot \left|F_{Z}\right|$$

where

$$\begin{array}{l} M_{14b}=M_{8b}\cdot M_{13b}+M_{9b}\\ M_{15b}=M_{8b}\cdot M_{11b}\\ M_{16b}=M_{8b}\cdot M_{12b} \end{array}$$

are known matrices if the motions of the mechanism are given. There are six equations and eight unknowns in τ and $F_{Z}$ in this model, indicating that actuating torques and friction effects should be optimally computed. Furthermore, a closer observation of Equation (50) can lead to the following remarks:

1. Even if a simple Coulomb and viscous friction model, which is classified as a static one in [39] is employed, friction effects at HKPs significantly enhance the nonlinearity of the dynamic model in terms of $\left|F_{Z}\right|$ explicitly, and they implicitly influence frictional torques $\tau_{f}$, since $\tau_{f}$ is the function of $\left|F_{Z}\right|$ as shown in Equations (39), (42), and (49). Regarding these, friction effects at HKPs and revolute joints are strongly coupled, and actuating torques are influenced by them.
2. If all friction effects are neglected, Equation (50) degrades to the form of

(51) $$\tau =M_{1}-M_{2}\cdot F_{Z}$$

where ***M***_1_ and ***M***_2_ are matrices that are the functions of kinematic and dynamic parameters of the target PM. Equation (51) is actually the simpler linear inverse dynamic model of the target mechanism free of friction with six equations and eight unknowns, including actuations and constraint forces ***F****_Z_*. The form of Equation (51) is very different from that of the final EOMs of PMs in [31,32,33], since their unknowns are only actuations.

### 4.5. Optimal Goals to Distribute Actuating Torques

Because the inverse dynamic model of the mechanism under study is underdetermined with six nonlinear equations and eight unknowns, actuating torques can be optimally distributed to meet different dynamic performances. In this optimisation problem, the eight unknowns are the optimal variables, and physical constraints include:

Constraint forces FGiCi0 cannot exceed the axial and radial loading capacities of the chosen actuator in the prototype;

The output power of the actuator is below its maximum power capacity;

The inverse dynamic model, Equation (50), is the nonlinear equality constraint.

Based on the physical application of the mechanism, three optimal goals are individually set to produce different performances.

As far as the initial guess under the three optimal goals is concerned, at *t* = 0, $\tau =0_{6\times 1}$ and $F_{Z}=0_{2\times 1}$ are used as the initial guess, and the obtained values of τ and ***F****_Z_* are used as the initial guess of the optimisation scheme at the next time instant. This loop is repeated for each time interval until the end of the timeline.

#### 4.5.1. Minimal Actuating Torques

This goal can be mathematically defined as

(52) $$G_{1}=\min\left\|\tau \right\|$$

where $\left\|\tau \right\|$ is the two-norm sum of τ. The physical meaning of this goal is to minimize the output torque from the actuator, in favour of motor sizing in the design stage of the mechatronics system. In fact, this goal is often achieved in PMs with redundant actuations by virtue of the Moore–Penrose pseudo-inverse matrix to directly compute τ, as in [40,41,42,43]. However, in Equation (50), τ and $F_{Z}$ have different units, and the nonlinear term $M_{16b}\cdot \left|F_{Z}\right|$ exists. Henceforth, a more complex optimisation algorithm is needed to numerically compute τ and $F_{Z}$ simultaneously.

#### 4.5.2. Minimal Constraint Forces at S_1_~S_6_

As shown from Equations (38) and (46), $F_{S_{i}}$ directly influences constraint wrenches at *G_i_*. Because the chewing behaviours of human beings to be mimicked by the designed mechanism are approximately periodic, large constraint wrenches at *G_i_* bring large vibrations and impulses to the base and neighbouring devices. As such, a second goal is set as

(53) $$G_{2}=\min\left\|F_{S_{1\_6}}\right\|$$

which can minimize the constraint forces at *S_i_* and then reduce these abovementioned negative effects.

#### 4.5.3. Minimal Constraint Forces at HKPs

A large constraint force at a HKP tends to cause large friction and then wear and tear easily occurs in the condylar ball and the condylar socket. Regarding this, a third goal is defined as

(54) $$G_{3}=\min\left\|F_{Z}\right\|$$

which can minimise the constraint forces at HKPs; thus, wear and clearance caused by friction effects can be minimised.

Finally, the sequential quadratic programming (SQP) method is characterised by fast convergence, and nonlinear constraints, as mentioned above, can be easily incorporated. Thus, this method is to be employed to optimally compute the dynamic model.

It is noted that what we are concerned about is whether the designed mechanism can vividly reproduce the chewing behaviours of human subjects; thus, this robotic device can be applied in the food industry to evaluate the newly developed food properties as mentioned in Section 1. Secondly, the mechanism is actually a simplified model of the human chewing system, which has more muscles than those in the designed mechanism. In these regards, how the muscles in the human masticatory system work synchronously under the control of the central neural system is left to the oral biologists to discover.

## 5. Numerical Computations and Discussions

The coordinates of *G_i_* and *S_i_* (*i* = 1,…,6) in frame {*S*}, and *M_i_* in frame {*M*} are summarised in Table 1, and the geometrical and inertia parameters of the mechanism are summarised in Table 2.

### 5.1. Computation of $X_{EE}^{P},{\dot{X}}_{EE}^{P},{\ddot{X}}_{EE}^{P}$ and $q_{EE}^{P},{\dot{q}}_{EE}^{P},{\ddot{q}}_{EE}^{P}$

To study the dynamic model numerically, the mechanism is commanded to follow a lower incisor path of a healthy volunteer in ${\mathbb{R}}^{3}$. To track the trajectory with respect to displacement, only three scalar equations can be formatted as

**Figure 5:**
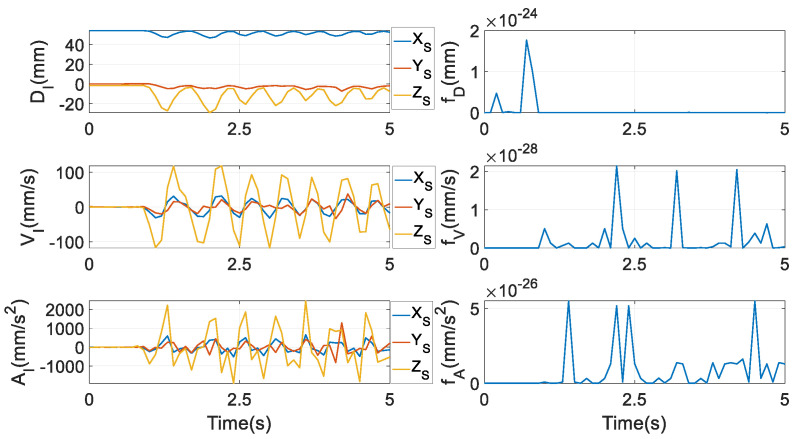
An incisor trajectory of a healthy human subject and the tracking errors.

(55) OSOM+RMSθ⋅OMMI−OMI=03×1

where OMMI contains three position coordinates of the incisor point *I* in frame {*M*}, and $O_{M}I$ is the position vector of this point in {*S*}, whose numerical values are exhibited in the first subplot of Figure 5. The letters D, V, and A in labels of the three subplots in the first column denote displacement, velocity, and acceleration, respectively. In Equation (55), there are only three equations but five unknowns in $q_{EE}^{P}$, i.e., theoretically, there are infinite solutions. Nonetheless, it is not easy to numerically resolve this set of nonlinear equations. From the literature, the algorithm in [44] enlightened us: to identify the solution to a transcendental equation in that paper, the equation is not computed numerically. The researchers’ logic is that the values of the unknown variables that can make the absolute value of the transcendental equation as small as possible are the solutions. By virtue of this idea, to find one feasible solution of $q_{EE}^{P}$, a single-aim optimisation problem is constructed as

(56) Aim:min fD=OSOM+RMSθ⋅OMMI−OMIUnknowns:qEEPRange of variables:−10≤X≤5−3≤Y≤3θi≤1, i=0,…,3

Constraints: Equations (5) and (7)

Method: SQP

The physical meaning of the aim is to track the predefined incisor trajectory with the smallest tracking error in terms of displacement. The ranges of *X* and *Y* are determined by the length and width of the socket that holds the condylar ball in the prototype, respectively.

After $q_{EE}^{P}$ is obtained, $X_{EE}^{P}$ can be computed by Equation (7). At *t* = 0, $q_{EE}^{P}=0_{5\times 1}$ is used as initial guess, and the obtained values of $q_{EE}^{P}$ are used as the initial guess of the optimisation scheme at the next time instant. The loop is repeated for each time interval until the end of the timeline.

Correspondingly, in tracking the velocity of this trajectory, ${\dot{q}}_{EE}^{P}$ cannot be uniquely determined, since, likewise, there are five unknowns in ${\dot{q}}_{EE}^{P}$ but only three equations, as

(57) $$M_{I}\cdot {\dot{q}}_{EE}^{P}-V_{I}=0_{3\times 1}$$

where ***M****_I_* is the 3 × 5 Jacobian matrix between the coordinates of the incisor point *I* and $q_{EE}^{P}$, and $V_{I}$ is the 3 × 1 velocity vector of the incisor trajectory, whose numerical values are given in the second subplot of the first column of Figure 5. From Equation (5), constraint equations from the velocity and the acceleration levels can be attained by differentiating it with respect to time once and twice, respectively

(58) $${\dot{\theta }}^{T}\cdot \theta =0.$$

(59) $$\left[\begin{array}{cc} {\ddot{\theta }}^{T} & {\dot{\theta }}^{T} \end{array}\right]\cdot \left[\begin{array}{c} \theta \\ \dot{\theta } \end{array}\right]=0$$

Thus, to reach one feasible solution of ${\dot{q}}_{EE}^{P}$, a second optimisation problem is set as

(60) $$\begin{array}{l} \mathrm{Aim}:\min\;f_{V}=\left\|M_{I}\cdot {\dot{q}}_{EE}^{P}-V_{I}\right\|\\ \mathrm{Unknowns}:{\dot{q}}_{EE}^{P} \end{array}$$

Constraints: Equations (10) and (58)

Method: SQP

where numerical values of $q_{EE}^{P}$ in ***M****_I_* are fed from those computed by Equation (56). The physical meaning of this aim is to track the predefined incisor trajectory with the smallest tracking error in terms of velocity. At *t* = 0, ${\dot{q}}_{EE}^{P}=0_{5\times 1}$ is used asan initial guess, and the obtained values of ${\dot{q}}_{EE}^{P}$ are used as the initial guesses of the optimisation scheme at the next time instant. The loop is repeated for each time interval until the end of the timeline.

Finally, to compute ${\ddot{q}}_{EE}^{P}$, only three scalar equations can be written as

(61) $$M_{I}\cdot {\ddot{q}}_{EE}^{P}+{\dot{M}}_{I}\cdot {\dot{q}}_{EE}^{P}-{\dot{V}}_{I}=0_{3\times 1}$$

where ${\dot{V}}_{I}$ is the predefined 3 × 1 acceleration vector of the incisor point *I*, whose numerical values are shown in the third subplot of the first column of Figure 5, and ${\dot{M}}_{I}$ is the first time-rate of $M_{I}$. Identically, to reach a feasible solution of ${\ddot{q}}_{EE}^{P}$, a third optimisation problem is set as

(62) $$\begin{array}{l} \mathrm{Aim}:\min\;f_{A}=\left\|M_{I}\cdot {\ddot{q}}_{EE}^{P}+{\dot{M}}_{I}\cdot {\dot{q}}_{EE}^{P}-{\dot{V}}_{I}\right\|\\ \mathrm{Unknowns}:{\ddot{q}}_{EE}^{P} \end{array}$$

Constraints: Equations (11) and (59)

Method: SQP

where numerical values of $q_{EE}^{P},{\dot{q}}_{EE}^{P}$ in ***M****_I_* and ${\dot{M}}_{I}$ are fed from those computed in Equations (56) and (60). The physical meaning of this aim is to track the predefined incisor trajectory with the smallest tracking error in terms of acceleration. After this, ${\dot{X}}_{EE}^{P}$ and ${\ddot{X}}_{EE}^{P}$ can be computed from Equation (10) and Equation (11), respectively. At *t* = 0, ${\ddot{q}}_{EE}^{P}=0_{5\times 1}$ is used as initial guess, and the obtained values of ${\ddot{q}}_{EE}^{P}$ are used as the initial guess of the optimisation scheme at the next time instant. The loop is repeated for each time interval until the end of the timeline.

**Figure 6:**
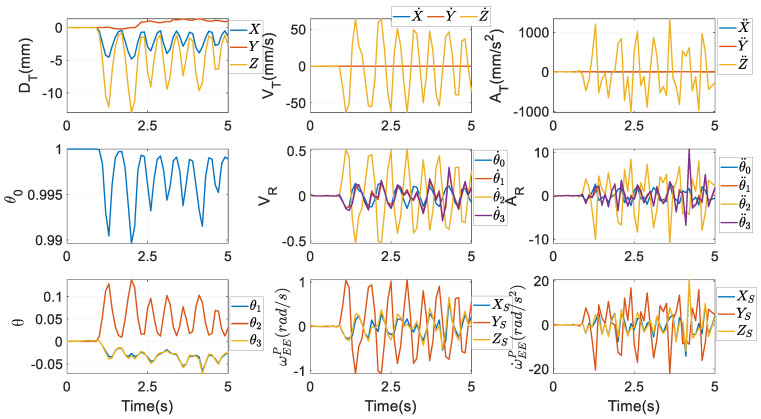
Motions of the end effector by $X_{EE}^{P},{\dot{X}}_{EE}^{P},{\ddot{X}}_{EE}^{P}$.

By these three optimisation procedures, the numerical values of *f_D_*, *f_V_*, and *f_A_* are obtained as in the second column of Figure 5, being very tiny. Thus, the predefined incisor trajectory is reckoned to be tightly followed in terms of displacement, velocity, and acceleration. Correspondingly, all $X_{EE}^{P},{\dot{X}}_{EE}^{P},{\ddot{X}}_{EE}^{P}$ are shown in the first seven subfigures of Figure 6, where D, V, and A mean displacement, velocity, and acceleration, respectively, and the subscripts T and R indicate translation and rotation, respectively. Unambiguously, the motion range of the parasitic motion variable *Z* is far larger than that of *X* and *Y*, and the magnitudes of $\dot{Z}$ and $\ddot{Z}$ are also far larger than their counterparts. This discovery is very different from [45], where parasitic motions are required to be as small as possible. Additionally, the magnitudes of *θ*_1_ and *θ*_3_ are nearly equivalent, due to the coefficient in front of *θ*_1_ in the formula of *θ*_3_ from Equation (7) being almost equal 1. $\omega_{EE}^{P}$ and ${\dot{\omega }}_{EE}^{P}$ are computed by Equations (12) and (14), and they are given in the last two subfigures.

.

Note that by following the predefined incisor trajectory, the denominator of *θ*_3_ as in Equation (7), is nonzero. Perhaps in the entire workspace of the mechanism, there are some configurations where the denominator of *θ*_3_ is zero; then *θ*_3_ cannot be computed from Equation (7), and probably some other coordinates in the four Euler parameters would be switched as parasitic motion variables. It is worth a deeper investigation in the future work.

### 5.2. Transformation Between Euler Parameters and Euler Angles

A critical reason to employ Euler parameters to describe the constrained motions of the end effector is to reduce the computational cost, as stated in Section 1. To this end, for a fair comparison, the end effector must undergo identical motions as expressed by Euler parameters if some other sets of parameters are employed, such as Euler angles. However, this cannot be realised in the target PM due to DCFB. The reason is as follows.

Firstly, when Euler angles are used to describe the rotation of the end effector, the parameters describing its configuration are grouped in a 6 × 1 vector as in Equation (3) of [20]

(63) $$X_{EE}^{A}={\left[\begin{array}{cccccc} X^{A} & Y^{A} & Z^{A} & \alpha & \beta & \gamma \end{array}\right]}^{T}$$

where *X^A^*, *Y^A^*, and *Z^A^* are the coordinates of *O_M_* in the inertia frame {*S*}, and *α*, *β*, and *γ* are the *XYZ* Euler angles. A superscript *A* is added to indicate parameters expressing translations are used together with Euler angles. Then, the four DOFs are grouped in a 4 × 1 vector as

(64) $$q_{EE}^{A}={\left[\begin{array}{cccc} X^{A} & Y^{A} & \alpha & \beta \end{array}\right]}^{T}$$

For the parasitic motions, *Z^A^* owns a completely identical expression as *Z* in Equation (7); however, the rotation matrix RMS in it is expressed by Euler angles. The parasitic Euler angle γ in Equation (5) of [20] is repeated here as

(65) $$\gamma =-\mathrm{atan}\left(\frac{s\alpha }{p_{1}c\beta +c\alpha s\beta }\right)$$

where *s*(·) and *c*(·) indicate *sin*(·) and *cos*(·), respectively.

Thereby, by virtue of Euler angles, these parasitic motions are full of trigonometric functions. Note that two translational DOFs along the *Xs* and *Ys* axes of {*S*} exist in both $q_{EE}^{A}$ and $q_{EE}^{P}$. As such, to derive the passage from Euler parameters to Euler angles, one can easily define

(66) XA=XYA=YRMSθ=RMSα,β,γ

where RMSα,β,γ is the rotation matrix calculated by *α*, *β*, and *γ*. Then, an identical posture of the end effector in terms of both the translation of *O_M_* and the orientation of the end effector can be achieved by Euler parameters and Euler angles. The three Euler angles can be computed by

(67) $$\begin{array}{l} \alpha =\mathrm{atan}2\left(-r_{23}/c\beta ,\;r_{33}/c\beta \right)\\ \beta =\mathrm{asin}\left(r_{13}\right)\\ \gamma \;\mathrm{in}\;\mathrm{Equation}\;(65)\; \end{array}$$

where *r_ij_* (*i*, *j* = 1,…,3) means the element at the *i*th row and *j*th column of the rotation matrix RMSθ in Equation (4).

However, one identical twist of the end effector is not easy to be reached by these two sets of parameters. Apart from Equation (12), the twist using ${\dot{q}}_{EE}^{A}$ can also be written in the form of

(68) $$t_{EE}=M_{A}\cdot {\dot{q}}_{EE}^{A}$$

where $M_{A}$ is the 6 × 4 twist-shaping matrix and ${\dot{q}}_{EE}^{A}$ is the first time-rate of $q_{EE}^{A}$.

If ***t****_EE_* from Equations (12) and (68) is equivalent, we can find

(69) $$t_{EE}=M_{1b}\cdot {\dot{q}}_{EE}^{P}=\left[\begin{array}{cc} I_{2} & O_{2\times 3}\\ M_{1b2} & \end{array}\right]\cdot \left[\begin{array}{c} \dot{X}\\ \dot{Y}\\ {\dot{\theta }}_{0}\\ {\dot{\theta }}_{1}\\ {\dot{\theta }}_{2} \end{array}\right]=M_{A}\cdot {\dot{q}}_{EE}^{A}=\left[\begin{array}{cc} I_{2} & O_{2}\\ M_{A2} & \end{array}\right]\cdot \left[\begin{array}{c} {\dot{X}}^{A}\\ {\dot{Y}}^{A}\\ \dot{\alpha }\\ \dot{\beta } \end{array}\right]$$

where ***I***_2_ is the 2 × 2 identity matrix, $O_{2\times 3}$ and ***O***_2_ are the 2 × 3 and 2 × 2 zero matrix, respectively, ***M***_1*b*2_ is the 4 × 5 submatrix of ***M***_1*b*_ containing its third to the sixth rows, and ***M****_A_*_2_ is the 4 × 4 submatrix of ***M****_A_* containing its third to the sixth rows. Thus, from the first two rows of Equation (69), we can find

(70) $$\begin{array}{c} {\dot{X}}^{A}=\dot{X}\\ {\dot{Y}}^{A}=\dot{Y} \end{array}$$

In fact, this can also be attained from the first two equations of Equation (66). Afterwards, from the last four rows, we can obtain

(71) $$M_{1b21}\cdot \left[\begin{array}{c} \dot{X}\\ \dot{Y} \end{array}\right]+M_{1b22}\cdot \left[\begin{array}{c} {\dot{\theta }}_{0}\\ {\dot{\theta }}_{1}\\ {\dot{\theta }}_{2} \end{array}\right]=M_{A21}\cdot \left[\begin{array}{c} {\dot{X}}^{A}\\ {\dot{Y}}^{A} \end{array}\right]+M_{A22}\cdot \left[\begin{array}{c} \dot{\alpha }\\ \dot{\beta } \end{array}\right]$$

where $M_{1b2}=\left[\begin{array}{cc} \underset{4\times 2}{\underset{︸}{M_{1b21}}} & \underset{4\times 3}{\underset{︸}{M_{1b22}}} \end{array}\right]$ and $M_{A2}=\left[\begin{array}{cc} \underset{4\times 2}{\underset{︸}{M_{A21}}} & \underset{4\times 2}{\underset{︸}{M_{A22}}} \end{array}\right]$. It derives that

(72) $$\left(\underset{4\times 2}{\underset{︸}{M_{1b21}}}-\underset{4\times 2}{\underset{︸}{M_{A21}}}\right)\cdot \left[\begin{array}{c} \dot{X}\\ \dot{Y} \end{array}\right]+\underset{4\times 3}{\underset{︸}{M_{1b22}}}\cdot \left[\begin{array}{c} {\dot{\theta }}_{0}\\ {\dot{\theta }}_{1}\\ {\dot{\theta }}_{2} \end{array}\right]=\underset{4\times 2}{\underset{︸}{M_{A22}}}\cdot \left[\begin{array}{c} \dot{\alpha }\\ \dot{\beta } \end{array}\right]$$

If the twist of the end effector ***t****_EE_* is predefined from $M_{1b}\cdot {\dot{q}}_{EE}^{P}$, i.e., Equation (12), then we need to compute $\dot{\alpha }$ and $\dot{\beta }$ from Equation (72); however, the four rows of $M_{A22}$ are generally independent. Thus, it is an overdetermined set of linear equations. It is not easy to find the solutions to $\dot{\alpha }$ and $\dot{\beta }$. Likewise, if ***t****_EE_* is predefined from $M_{A}\cdot {\dot{q}}_{EE}^{A}$, i.e., Equation (68), then we need to compute ${\dot{\theta }}_{0}$~${\dot{\theta }}_{2}$ from Equation (72), and generally the four rows of ***M***_1*b*22_ are independent. Thus, it is not easy to find the solutions to ${\dot{\theta }}_{0}$~${\dot{\theta }}_{2}$ neither.

Following the same logic, a first time-rate of the twist using Euler parameters cannot be equivalently expressed by Euler angles, and vice versa. This phenomenon is clearly caused by DCFB which produce parasitic motions and then reduce the number of DOFs. In summary, an identical configuration can be reached by Euler parameters and Euler angles, whilst these two sets of parameters can reach neither an identical twist nor its first time-rate.

**Figure 7:**
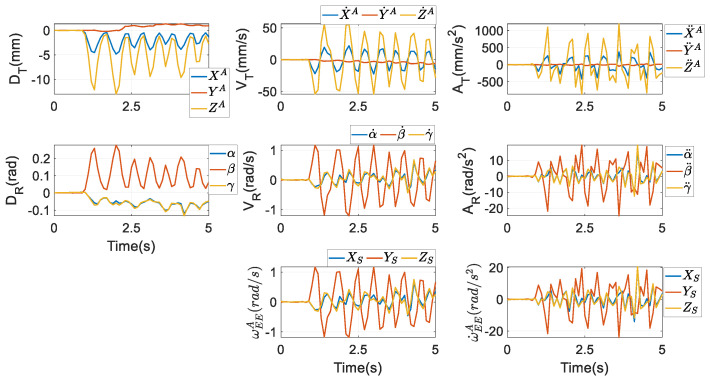
Motions of the end effector by $X_{EE}^{A},{\dot{X}}_{EE}^{A},{\ddot{X}}_{EE}^{A}$.

In this regard, the optimisation procedure in Section 5.1 is implemented again to obtain $q_{EE}^{A},{\dot{q}}_{EE}^{A},{\ddot{q}}_{EE}^{A}$, which are independent of the procedures to compute $q_{EE}^{P},{\dot{q}}_{EE}^{P},{\ddot{q}}_{EE}^{P}$. Then, from Section 3 of [20], $X_{EE}^{A},{\dot{X}}_{EE}^{A},{\ddot{X}}_{EE}^{A}$ and $\omega_{EE}^{A}$ can be computed as given in Figure 7, and ${\dot{\omega }}_{EE}^{A}$ can be further attained by differentiating $\omega_{EE}^{A}$. These values are used in the dynamic model to make a relatively fair comparison, to find which set of parameters is more computationally efficient.

From Figure 6 and Figure 7, their first subplots are equivalent, as stated after Equation (66). However, as far as the first time-rate of the coordinates of the mass centre *O_M_* is concerned, from the second subplot, differences between $\dot{X}$ and ${\dot{X}}_{A}$, and those between $\dot{Y}$ and ${\dot{Y}}_{A}$ are much more apparent. The same conclusions can also be made in terms of the second time-rate of the coordinates of *O_M_*, as shown in the third subplot. From the subplots about rotations, though the profiles of *θ*_1_~*θ*_3_ are very close to those of *α*, *β*, and *γ*, their amplitudes are clearly not equivalent. Through the optimisation scheme in Equation (56), the coefficient in front of *θ*_1_ in the formula of *θ*_3_ from Equation (7) almost equals 1. Besides, the analogous optimisation scheme also renders the value of *γ* almost equivalent to that of *α* through Equation (64).

**Figure 8:**
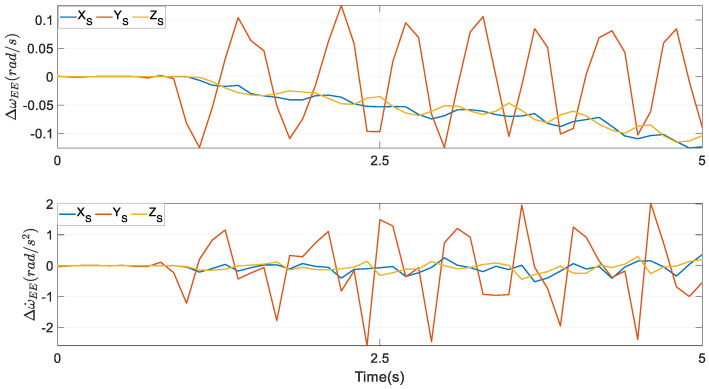
Angular velocity and angular acceleration differences were computed from Euler parameters and Euler angles in the end effector of the target mechanism.

The profiles of the rotational velocity $\omega_{EE}^{P}$ computed via Euler parameters at the bottom row of Figure 6 are similar to those of the rotational velocity $\omega_{EE}^{A}$ computed via Euler angles at the bottom row of Figure 7. Actually, their numerical values are different at every time instant, however. The same conclusion can be reached by the rotational acceleration. The differences between $\omega_{EE}^{A}$ and $\omega_{EE}^{P}$ are displayed in the first subplot of Figure 8, while in the second one, the differences between ${\dot{\omega }}_{EE}^{A}$ and ${\dot{\omega }}_{EE}^{P}$ are presented.

### 5.3. Dynamic Performances

Corresponding to the three optimal goals in Section 4.5, the following performance indices are set to justify the optimisation problem:

(73) $$\begin{array}{l} F_{1}=\frac{1}{N}\sum_{i=1}^{N}{\left\|\tau \right\|}_{i}\;,\;F_{3}=\frac{1}{N}\sum_{i=1}^{N}{\left\|F_{Z}\right\|}_{i}\\ F_{2}=\frac{1}{N}\sum_{i=1}^{N}{\left\|F_{S_{1\_6}}\right\|}_{i} \end{array}$$

where *N* is the number of sampling instants along the timeline, ${\left\|\cdot \right\|}_{i}$ is the two-norm sum of the specific vector at the *i*th instant. The physical meanings of *F*_1_~*F*_3_ are to compute the mean values of the two-norm sum of the actuating torques, the constraint forces at HKPs along the *Z_S_* axis, and the constraint forces at *S_i_* (*i* = 1,…,6), respectively, along the timeline. They correspond to the three optimal goals in Section 4.5 sequentially.

Additionally, some other indices are set to see their strong correlations with *F*_2_ and *F*_3_

(74) $$\begin{array}{l} F_{4}=\frac{1}{N}\sum_{i=1}^{N}{\left\|\tau_{f}\right\|}_{i}\;,\;\;\;F_{6}=\frac{1}{N}\sum_{i=1}^{N}{\left\|f_{T_{L}}+f_{T_{R}}\right\|}_{i}\\ F_{5}=\frac{1}{N}\sum_{i=1}^{N}{\left\|M_{G_{1\_6}}^{C}\right\|}_{i}\;,\;\;F_{7}=\frac{1}{N}\sum_{i=1}^{N}{\left\|F_{G_{1\_6}}\right\|}_{i} \end{array}$$

where MG1_6C=MG1S1CC10⋮MG6S6CC60. The physical meanings of *F*_4_~*F*_7_ are to compute the mean values of the two-norm sum of the friction torques at active revolute joints, the constraint moments at *G_i_* (*i* = 1,…,6), the sum of the friction forces at two HKPs, and the constraint forces at *G_i_* (*i* = 1,…,6), respectively, along the timeline. Evidently, from the derivation of the dynamic model in Section 4.1, Section 4.2, Section 4.3 and Section 4.4, *F*_4_~*F*_6_ and *F*_7_ are tightly related to *F*_2_ and *F*_3_, respectively. By setting these performance indices, the correctness of the computations can be verified.

In general, true friction coefficients in the friction model are identified in practice to compensate for their effects; in this paper, their values are assumed to show the friction effects, however. Their practical identification will be performed in the future. For the Coulomb and viscous friction model, all the coefficients are set as

$$\begin{array}{l} \mu_{C}=0.02,\;\;\mu_{V}=0.03g/s,\;\;\\ \mu_{C_{i}}=0.02,\;\;\mu_{V_{i}}=0.03g/s,\;\;R_{i}=15\mathrm{mm}\left(i=1,\ldots ,6\right) \end{array}$$

**Figure 9:**
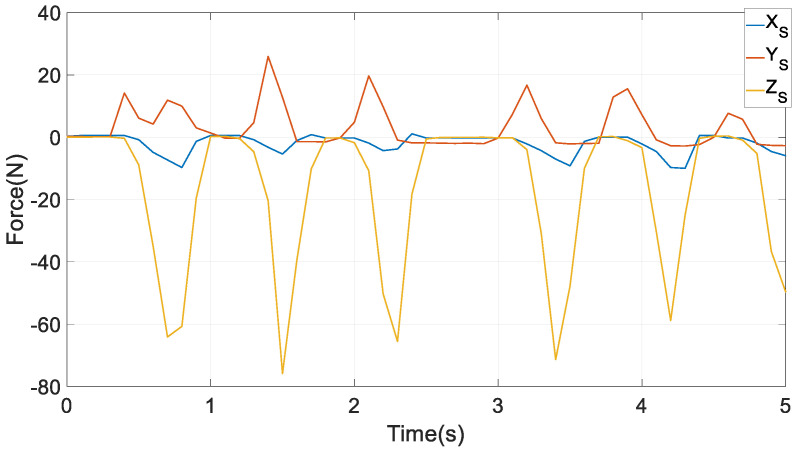
Reaction forces from chewing on peanuts to the molar in {*S*} [20].

An experimentally measured reacted chewing force in {*S*} on peanuts by an orally healthy male volunteer on his molars, as in Figure 9, acts on the lower left molar at point *B*. The magnitude in the vertical direction in the inertia frame is far larger than its components along the *X_S_* and *Y_S_* axes in every stroke, indicating that larger bite forces in this direction are needed to chew the peanuts.

**Figure 10:**
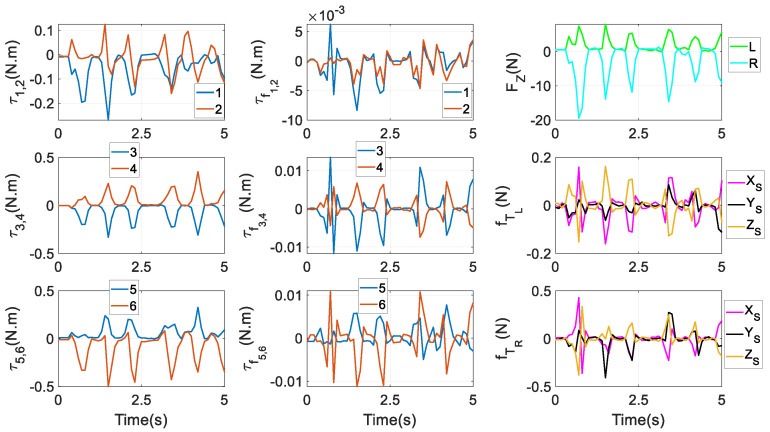
Actuating torques and friction torques at active revolute joints, and constraint forces and friction forces at HKPs under the first optimal goal in Equation (52).

Under the first aim in Equation (52), actuating torques τ, friction torques $\tau_{f}$, constraint forces ***F****_Z_*, and friction forces $f_{T_{i}}$ at *T_i_* are given in Figure 10. All these variables are following an identical rhythm. Evidently, there is a certain degree of symmetry between $\tau_{i}$ and $\tau_{i+1}$, $\tau_{f_{i}}$ and $\tau_{f_{i+1}}$ (*i* = 1,3,5), respectively. At most of the time instants, $F_{Z_{R}}$ and $F_{Z_{L}}$ are negative and positive, respectively, indicating that the right and left condylar balls are receiving constraint forces from the upper and lower surfaces of the two condylar sockets, respectively. That is because a reacted bite force ***F****_B_* is acting at a left molar, tending to rotate the end effector around the positive direction of the *X_S_* axis. Additionally, $F_{Z_{R}}$ has larger peaks than $F_{Z_{L}}$, then friction peaks at *T_R_* are accordingly larger than those at *T_L_*.

The proportions between friction effects and actuating torques in each actuator are computed as

(75) $$\begin{array}{cc} P_{i}=\frac{\tau_{F_{i}}}{\tau_{i}} & \left(i=1,\ldots ,6\right) \end{array}$$

where $\tau_{F_{i}}$ is the *i*th entry of $\tau_{F}$, and $\tau_{F}$ is the 6 × 1 friction torque vector accounting for friction effects at both HKPs and actuators with respect to the output shafts of actuators.

**Figure 11:**
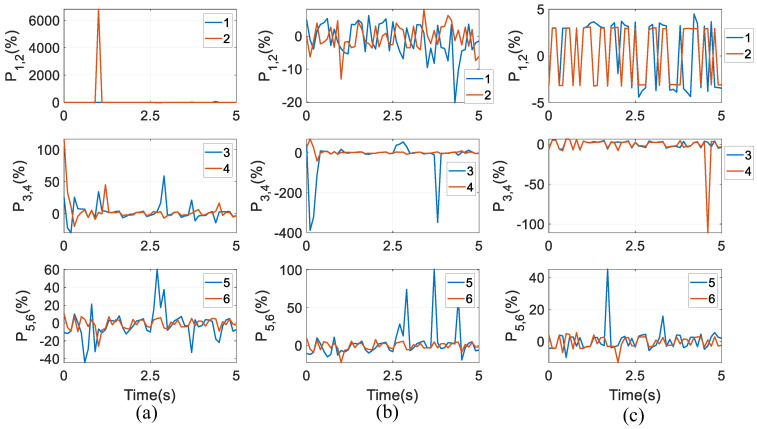
Proportions between friction effects and actuating torques under three optimal goals: (**a**) the first goal, (**b**) the second goal, and (**c**) the third goal.

Numerical results under the three optimal goals are displayed in the three columns of Figure 11, respectively. Under the first aim, the proportion in the second actuator can even reach up to over 6000% at *t* = 1 s. The largest proportion in the fourth actuator is over 100% at *t* = 0, and at *t* = 2.7 s, the largest proportion in the fifth actuator is approximately 60%. Under the second aim, one can also find that at *t* = 0.1 s and 3.8 s, the proportion in the fourth actuator is over 350%, and at *t* = 2.9 s, 3.7 s, and 4.4 s, the proportion in the fifth actuator is between 50% and 100%. Finally, under the third aim, at *t* = 4.6 s, the proportion in the fourth actuator is over 100%. This shows that the friction has negative effects that cannot be ignored in the motion accuracy, and lubrications are needed to reduce wear at HKPs and revolute joints. Additionally, the proportions vary significantly across different optimal goals for torque distributions. Under the third goal, in the first and second actuators, the proportions are limited between ±5%, being very consistent.

**Figure 12:**
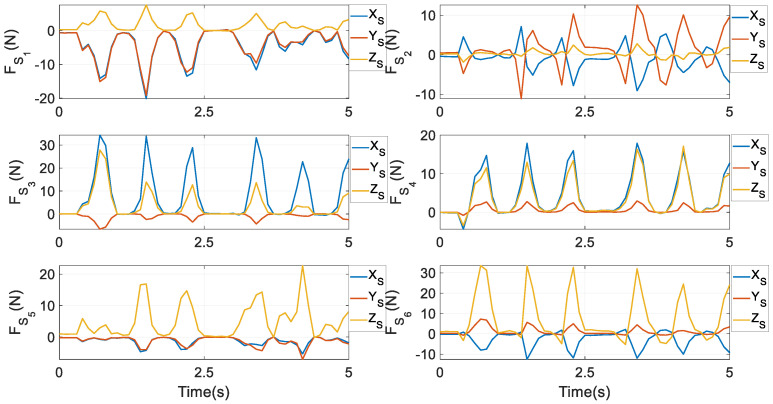
Constraint forces $F_{S_{i}}\left(i=1,\ldots ,6\right)$ under the first optimal goal in Equation (52).

**Figure 13:**
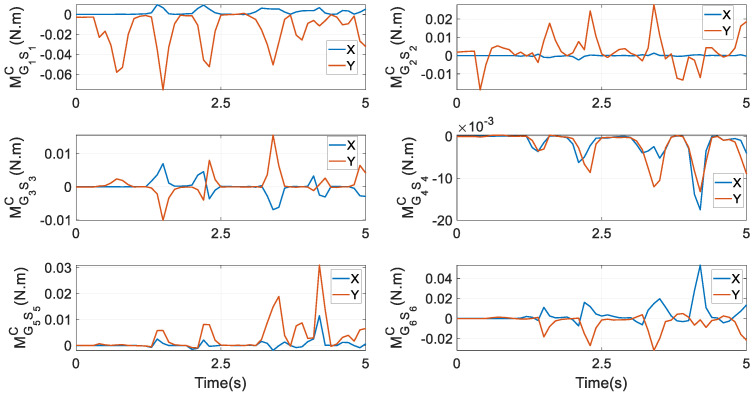
Constraint moments at *G_i_* (*i* = 1,…,6) under the first optimal goal in Equation (52).

Constraint forces at *S_i_* (*i* = 1,…,6) are given in Figure 12. It is interesting to notice that at *S*_1_, the components along the *X_S_* and *Y_S_* axes are nearly equivalent, whilst at *S*_2_, the component along the *Y_S_* axis has the highest peak. At *S*_3_ and *S*_4_, the component along the *X_S_* axis experiences the largest magnitude, while at *S*_5_ and *S*_6_, the component along the *Z_S_* axis has the largest magnitude. In Equation (37), from $F_{S_{i}}$ to constraint forces $F_{G_{i}}$ at *G_i_*, only one term $m_{G_{i}S_{i}}\cdot g$ is needed; while in the prototype, the crank is not heavy. As a result, the graphical exhibitions of $F_{S_{i}}$ and $F_{G_{i}}$ do not have a significant difference. The figure of $F_{G_{i}}$ is not provided to save pages. Constraint moments at *G_i_* around $X_{C_{i0}}$ and $Y_{C_{i0}}$ axes are given in Figure 13. By comparing it with the first column of Figure 10, apparently, the magnitude of constraint moments is much smaller than that of actuating torques. Under the second and third optimal goals, the profiles of actuating torques and friction torques at revolute joints, constraint forces and friction forces at *T_i_* (*i* = *L*, *R*), constraint forces at *S_i_* (*i* = 1,…,6), and constraint moments at *G_i_* are similar to their counterparts as in Figure 8, Figure 9, Figure 10 and Figure 11. Thus, they are not depicted as saving pages.

To make a comparison of dynamic performances under different optimal goals, numerical values of the proposed indices in Equations (73) and (74) are given in Table 3. The performance indices are always the smallest under the corresponding optimal goals, as remarked in bold, meaning the correctness of the optimisation issues in Section 4.5. Specifically, under *G*_3_, *F*_3_ is almost zero, so friction forces there can be sharply reduced, and a longer period of utilisation of HKP-related mechanical parts can be permitted. Additionally, due to the correlation between *F*_2_ and *F*_4_~*F*_6_, the smallest *F*_2_ under *G*_2_ also gives rise to the smallest *F*_4_~*F*_6_. Owing to the strong correlation between *F*_3_ and *F*_7_, the smallest *F*_3_ under *G*_3_ also produces the smallest *F*_7_. These numerical results prove the correctness of the computation. The inverse dynamic model using $q_{EE}^{A},{\dot{q}}_{EE}^{A},{\ddot{q}}_{EE}^{A}$ also reaches the identical remarks as above-mentioned, which can be found in the second row of Table 3.

**Figure 14:**
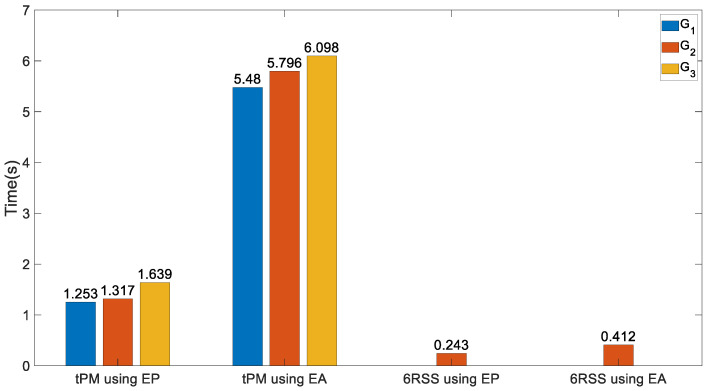
Computational time of the mechanism under study from three optimal goals and its counterpart without DCFB.

The procedures are formatted in Matlab installed on a personal computer with an Intel (R) Xeon (R) W-2235 CPU@3.80 GHz and 32 GB of RAM. The computational time of the target PM using $X_{EE}^{P},{\dot{X}}_{EE}^{P},{\ddot{X}}_{EE}^{P}$ and $X_{EE}^{A},{\dot{X}}_{EE}^{A},{\ddot{X}}_{EE}^{A}$ under three different optimal goals is given in the first two clusters of Figure 14. The dynamic model using Euler parameters to express motions of the end effector is much less time-consuming, which is only approximately 23% of that using Euler angles. Hence, the computational demands can be considerably alleviated by Euler parameters; then real-time control is possible. It apparently proves that in RMS and parasitic motion variables, the algebraic functions are more efficient than the trigonometric functions, generating a faster dynamic model. Additionally, the computational cost under the third optimal goal is a little heavier than that under the other two goals in the target PM.

### 5.4. The 6RSS PM

**Figure 15:**
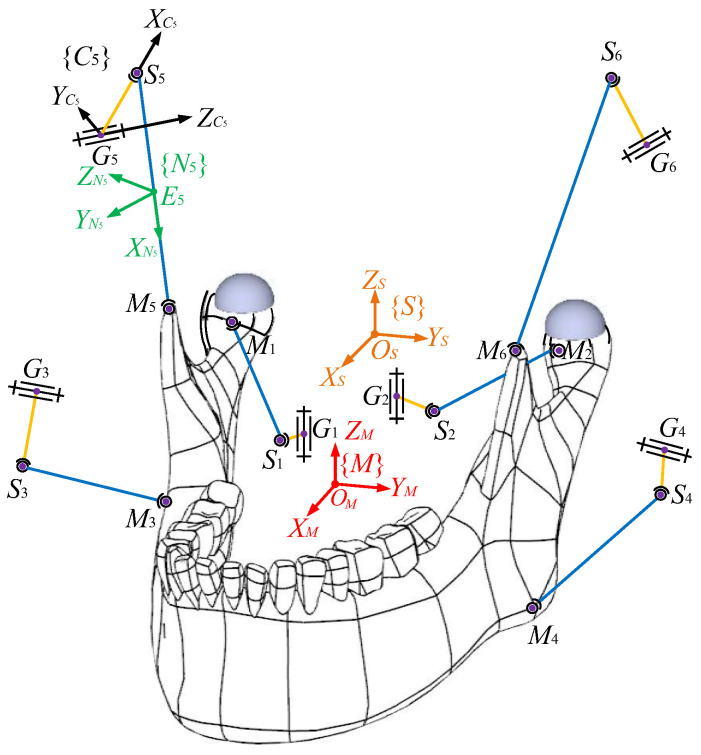
Schematic diagram of the 6RSS PM.

A schematic diagram of the 6RSS PM is displayed in Figure 15. From the comparison between it and Figure 1, one can see that clearly the 6RSS PM can be obtained by deleting the DCFB from the end effector, while other items are invariant.

As stated in Section 5.2, due to DCFB in the target mechanism, an identical twist and its first time-rate of the end effector cannot be achieved by $q_{EE}^{P},{\dot{q}}_{EE}^{P},{\ddot{q}}_{EE}^{P}$ and $q_{EE}^{A},{\dot{q}}_{EE}^{A},{\ddot{q}}_{EE}^{A}$, since DCFB eliminates DOFs and produces parasitic motions. However, in the 6RSS PM with three translational DOFs and three rotational DOFs, both the angular velocity and acceleration of its end effector using Euler parameters can be easily converted to those using Euler angles, and vice versa. The reason is as follows: The two sets of parameters to describe the configuration of the end effector are

(76) $$\begin{array}{l} Y_{EE}^{P}={\left[\begin{array}{cccc} x & y & z & e^{T} \end{array}\right]}^{T},\;\;\;\;e={\left[\begin{array}{cc} e_{0} & e \end{array}\right]}^{T},\;\;e={\left[\begin{array}{ccc} e_{1} & e_{2} & e_{3} \end{array}\right]}^{T}\\ Y_{EE}^{A}={\left[\begin{array}{cccc} x & y & z & \Phi^{T} \end{array}\right]}^{T},\;\;\Phi ={\left[\begin{array}{ccc} \phi_{1} & \phi_{2} & \phi_{3} \end{array}\right]}^{T} \end{array}$$

where *x*, *y*, and *z* are translational DOFs along the three axes in frame {*S*}, ***e*** is Euler parameters, and $\phi_{1}$, $\phi_{2}$, and $\phi_{3}$ are three *XYZ* Euler angles. Since its translations at position, velocity, and acceleration levels are completely independent of these two sets of parameters describing rotations, in the following, only the rotation at its three levels is analysed using Euler parameters and Euler angles. The angular velocity of the end effector using these two sets of parameters is expressed as

(77) $$\omega^{P}=2\cdot G\cdot \dot{e}$$

and

(78) $$\omega^{A}=R_{\omega }\cdot \dot{\Phi }$$

respectively, where

$$G=\left[\begin{array}{ll} -e, & e_{0}\cdot I_{3}+e\times \end{array}\right],\;R_{\omega }=\left[\begin{array}{lll} 1 & 0 & s\phi_{2}\\ 0 & c\phi_{1} & -s\phi_{1}c\phi_{2}\\ 0 & s\phi_{1} & c\phi_{1}c\phi_{2} \end{array}\right].$$

When the rotation is defined by ***e***, the three Euler angles can be computed as

(79) $$\begin{array}{l} \phi_{1}=\mathrm{atan}2\left(-s_{23}/c\phi_{2},\;s_{33}/c\phi_{2}\right)\\ \phi_{2}=\mathrm{asin}\left(s_{13}\right)\\ \phi_{3}=\mathrm{atan}2\left(-s_{12}/c\phi_{2},\;s_{11}/c\phi_{2}\right) \end{array}$$

where *s_ij_* (*i*, *j* = 1,…,3) means the element at the *i*th row and *j*th column of the rotation matrix defined by ***e***. According to Equations (77) and (78), when $\omega^{P}=\omega^{A}$ it yields

(80) $${\dot{Y}}_{EE}^{A}={R_{\omega }}^{-1}\cdot \omega^{P}$$

where $R_{\omega }$ is directly inverted if it is not singular. Specifically, when

(81) $$\left|R_{\omega }\right|=c\phi_{2}=0,$$

i.e., $\phi_{2}=\frac{\pi }{2}$, $R_{\omega }$ is singular, corresponding to the so-called gimbal lock inherent to Euler angles. However, this configuration can be circumvented in the trajectory planning. Specifically, when tracking the incisor trajectory in Figure 5, it is not reached, fortunately. Further, the angular acceleration by Euler parameters and Euler angles is

(82) $${\dot{\omega }}^{P}=2\cdot \left(\dot{G}\cdot \dot{e}+G\cdot \ddot{e}\right)$$

and

(83) $${\dot{\omega }}^{A}={\dot{R}}_{\omega }\cdot \dot{\Phi }+R_{\omega }\cdot \ddot{\Phi },$$

respectively. Thus, the second time-rate of Φ can be computed as

(84) $$\ddot{\Phi }=R_{\omega }^{-1}\cdot \left({\dot{\omega }}^{P}-{\dot{R}}_{\omega }\cdot \dot{\Phi }\right)$$

when ${\dot{\omega }}^{P}={\dot{\omega }}^{A}$.

On the contrary, if the rotation is defined by Euler angles Φ, one can have the values of **e** from Equations (47)–(50) of [46]

(85) $$e=\frac{1}{4e_{0}}\left[\begin{array}{c} 2e_{0}\sqrt{1+t_{11}+t_{22}+t_{33}}\\ t_{32}-t_{23}\\ t_{13}-t_{31}\\ t_{21}-t_{12} \end{array}\right]$$

where *t_ij_* (*i*, *j* = 1,…,3) means the element at the *i*th row and *j*th column of the rotation matrix defined by Φ. Because the four quantities in ***e*** are not all independent, from Equation (77), one can further write

(86) $$\omega^{P}=2\cdot \bar{G}\cdot \dot{e}$$

where $\bar{G}=G\cdot \left[\begin{array}{cc} 0_{1\times 3} & -\frac{e^{T}}{e_{0}}\\ 0_{3} & I_{3} \end{array}\right]$ and $\dot{e}$ means the first time-rate of **e**. Thus, all the three terms in $\dot{e}$ can be computed as

(87) $$\dot{e}=\frac{1}{2}\cdot {\bar{G}}^{-1}\cdot \omega^{A}$$

when $\bar{G}$ is invertible. The reason to choose *e*_0_ in the denominator in $\bar{G}$ is that in the workspace of the 6RSS PM, *e*_0_ is nonzero. Furthermore, when ${\dot{\omega }}^{P}={\dot{\omega }}^{A}$, from Equation (86), one can find

(88) $$\ddot{e}=\frac{1}{2}\cdot {\bar{G}}^{-1}\cdot \left({\dot{\omega }}^{A}-\dot{\bar{G}}\cdot \dot{e}\right)$$

Then, from

(89) $${\dot{e}}_{0}=-\frac{e^{T}}{e_{0}}\cdot \dot{e},\;{\ddot{e}}_{0}=-\frac{\left[\begin{array}{cc} {\dot{e}}^{T} & e^{T} \end{array}\right]\cdot \left[\begin{array}{c} \dot{e}\\ \ddot{e} \end{array}\right]}{e_{0}}$$

all four terms in $\dot{e}$ and $\ddot{e}$ are available numerically. Based on this derivation, for the end effector of the 6RSS PM, two sets of rotational parameters can be available to realise identical rotations at position, velocity, and acceleration levels.

**Figure 16:**
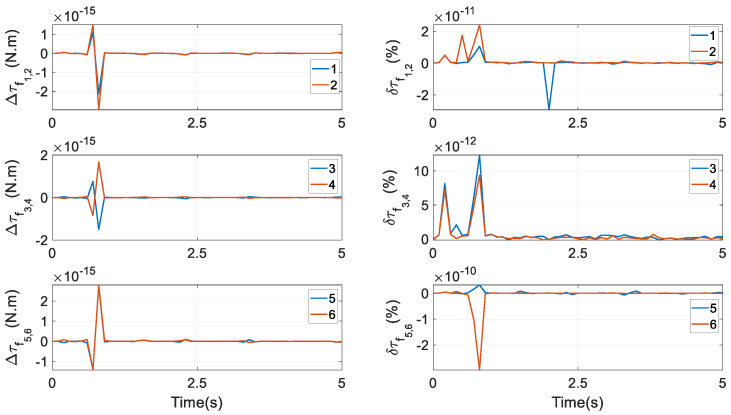
Differences and difference ratios of actuating torques of the 6RSS PM using Euler parameters and Euler angles.

In this regard, by giving the numerical values of $X_{EE}^{P},{\dot{X}}_{EE}^{P},{\ddot{X}}_{EE}^{P}$ of the target PM directly to $Y_{EE}^{P},{\dot{Y}}_{EE}^{P},{\ddot{Y}}_{EE}^{P}$ of the 6RSS PM, $Y_{EE}^{A},{\dot{Y}}_{EE}^{A},{\ddot{Y}}_{EE}^{A}$ can be computed using the procedure in this section, and the end effector of the 6RSS PM can perform identical motions as those of the end effector of the target PM, as shown in Figure 6, to make a fairer and more convenient comparison in computational demands between dynamic models of these two PMs. The inverse dynamic model of the 6RSS PM can be established following the procedure in Section 4; henceforth, it is not provided again. Note that because this mechanism has six actuations and six DOFs, its actuating torques have a closed-form solution without the optimisations in Section 4.5. On this basis, its dynamic performance is shown in the last two lines of Table 1, where the indices are completely identical. More precisely, the differences $\Delta_{\tau_{i}}$ and difference ratios $\delta_{\tau_{i}}$ of actuating torques are given in Figure 16, where

(90) $$\begin{array}{l} \Delta_{\tau_{i}}=\tau_{i}^{P}-\tau_{i}^{A},\left(i=1,\ldots ,6\right)\\ \delta_{\tau_{i}}=\frac{\tau_{i}^{P}-\tau_{i}^{A}}{\tau_{i}^{A}} \end{array}$$

$\tau_{i}^{P}$ and $\tau_{i}^{A}$ are the output torque by the *i*th actuator and calculated from the model using Euler parameters and Euler angles, respectively. These differences are very minor, indicating that under one identical motion expressed by these two sets of parameters, actuations are invariant. An identical exhibition can also be observed in friction torques at revolute joints. For the sake of brevity, their graph is not provided.

The computational time of the dynamic model of the 6RSS PM using Euler parameters is only approximately 60% of that using Euler angles, as shown in Figure 14, which again denotes that Euler parameters are more efficient than Euler angles. From the comparison between the mechanism under study and the 6RSS PM in Figure 14, DCFB significantly raises the modelling burden in kinematics and dynamics. Furthermore, one interesting discovery in Table 1 is, for the target PM under the third goal, i.e., Equation (54), all indices are close to those of the 6RSS PM, the constraint forces at HKPs being nearly zero, as if in the mechanism under study there were no DCFB to the end effector. Henceforth, the optimisation procedures play an important role in attaining it.

## 6. Conclusions

In this paper, the rotation of the end effector of a PM constrained directly by the base at two HKPs was expressed via Euler parameters, and the inverse dynamic model was built with friction effects at HKPs and active revolute joints using Newton–Euler’s law. Five conclusions can be drawn as follows:

1. The computational demand in the dynamic model with Euler parameters is only approximately 23% of that using Euler angles. Likewise, great computational savings can be achieved in the 6RSS PM. Thus, Euler parameters are an elegant alternative to Euler angles in releasing the nonlinearity and reducing the computational cost.
2. Euler parameters can be converted smoothly to Euler angles to realise identical configurations of the end effector of the PM under study. However, neither its twist nor the first time-rate of the twist can be set equivalent to these two sets of parameters. On the contrary, rotations of the end effector in the 6RSS PM can be set identically at the levels of angular displacement, velocity, and acceleration using Euler parameters and Euler angles. It is the DCFB that introduces these difficulties.
3. Friction effects at HKPs and revolute joints are strongly coupled via constraint forces at lower kinematic pairs as intermediate variables, and they significantly raise the nonlinearity of the dynamic model.
4. From the comparison between the mechanism under study and the 6RSS PM, even if DCFB is applied to the end effector, it considerably increases the computational demands in the former, actuating torques can be optimised to achieve different dynamic performances under different optimal goals. Performance indices that have strong relations with those under the predefined goals also reach their optimal values. By contrast, the inverse dynamic model of the 6RSS PM has a unique closed-form solution once its motions are predefined.
5. In the target PM, the computational cost under the third optimal goal is a little heavier than that under the other two goals, but this goal can facilitate a longer utilisation time of HKP-related mechanical parts. Under this goal, the performance indices of the target PM are almost equivalent to those of the 6RSS PM.

The dynamic model of the target mechanism using Euler parameters under the third goal will be incorporated into a motion controller design in experiments in our future work. Friction effects at HKPs and active joints will be identified offline and compensated for to raise the control accuracy.

## Figures and Tables

**Table 1:** Coordinates of *G_i_* and *S_i_* (*i* = 1,…,6) in frame {*S*}, and *M_i_* in frame {*M*} (unit: mm).

	*G*_1_	*G*_2_	*G*_3_	*G*_4_	*G*_5_	*G*_6_	*S*_1_	*S*_2_	*S*_3_	*S*_4_	*S*_5_	*S*_6_
*x*	23.65	23.65	40.15	40.15	36.15	36.15	32.19	32.19	54.11	54.11	23.96	23.96
*y*	−12.25	12.25	−58.24	58.24	−61.67	61.67	−17.45	17.45	−59.47	59.47	−63.49	63.49
*z*	−16.02	−16.02	−36.24	−36.24	39.47	39.47	−16.02	−16.02	−30.91	−30.91	48.02	48.02
	*M*_1_	*M*_2_	*M*_3_	*M*_4_	*M*_5_	*M*_6_						
*x*	10.33	10.33	28.61	28.61	36.13	36.13						
*y*	−40.47	40.47	−54.65	54.65	−52.46	52.46						
*z*	−7.00	−7.00	−51.28	−51.28	−1.32	−1.32						

**Table 2:** Mechanical parameters of the mechanism.

*p*_1_~*p*_6_ in Equation (2)	$\begin{array}{l} p_{1}=1.1,\;\\ p_{2}=-13.215\mathrm{mm},\;p_{3}=-10\mathrm{mm},\;p_{4}=5\mathrm{mm},\;p_{5}=69\mathrm{mm},\;p_{6}=75\mathrm{mm} \end{array}$
Mass of the end effector	*m_EE_* = 340.22 g
Inertia matrix of the end effector in {*M*}	IEEM=820,091.15−26.57−137,019.15−26.57423,459.18−88.60−137,019.15−88.60818,784.70 g⋅mm3
Lengths of couplers	$\left\|S_{i}M_{i}\right\|=33\;\mathrm{mm}\left(i=1,\ldots ,4\right),\left\|S_{j}M_{j}\right\|=52\;\mathrm{mm}\left(j=5,6\right)$
Inertia matrices of couplers in {*N_i_*}	IS1M1N1=IS2M2N2=IS3M3N3=IS4M4N4=diag12.331411.43411.43 g⋅mm2IS5M5N5=IS6M6N6=diag19.4311595.41595.4 g⋅mm2
Mass of cranks	$m_{G_{i}S_{i}}=70.2\;g\;\left(i=1,\ldots ,4\right),m_{G_{j}S_{j}}=156.69\;g\;\left(j=5,6\right)$
Rotational inertia of cranks	$I_{G_{i}S_{i}}=3510\;g\cdot {\mathrm{mm}}^{3}\;\left(i=1,\ldots ,4\right),\;I_{G_{j}S_{j}}=17,628\;g\cdot {\mathrm{mm}}^{3}\;\left(j=5,6\right)$
Radius of cranks	$\left\|G_{i}S_{i}\right\|=10\;\mathrm{mm}\;\left(i=1,\ldots ,4\right),\left\|G_{j}S_{j}\right\|=15\;\mathrm{mm}\;\left(j=5,6\right)$

**Table 3:** Dynamic performance indices in the target mechanism and the 6RSS PM.

		*F*_1_ (N.m)	*F*_2_ (W)	*F*_3_ (N)	*F*_4_ (N.m)	*F*_5_ (N.m)	*F*_6_ (N)	*F*_7_ (N)
Target PM with $q_{EE}^{P},{\dot{q}}_{EE}^{P},{\ddot{q}}_{EE}^{P}$	*G*_1_	**0.1912**	18.5039	4.2544	0.0219	0.0059	20.0250	0.1265
	*G*_2_	0.1958	**17.8948**	3.4370	**0.0184**	**0.0058**	**19.5470**	0.1022
	*G*_3_	0.2653	27.3216	**5.28 × 10^−6^**	0.0482	0.0084	28.4067	**1.64 × 10^−6^**
Target PM with $q_{EE}^{A},{\dot{q}}_{EE}^{A},{\ddot{q}}_{EE}^{A}$	*G*_1_	**0.1907**	18.4899	4.2236	0.0218	0.0059	20.0041	0.1256
	*G*_2_	0.1951	**17.9127**	3.4374	**0.0184**	**0.0058**	**19.5585**	0.1022
	*G*_3_	0.2638	27.2420	**1.75 × 10^−5^**	0.0481	0.0084	28.3257	**3.19 × 10^−6^**
6RSS PM with $Y_{EE}^{P},{\dot{Y}}_{EE}^{P},{\ddot{Y}}_{EE}^{P}$		0.2652	27.3214	-	0.0482	0.0084	28.4066	-
6RSS PM with $Y_{EE}^{A},{\dot{Y}}_{EE}^{A},{\ddot{Y}}_{EE}^{A}$		0.2652	27.3214	-	0.0482	0.0084	28.4066	-

## Data Availability

The data are available from the corresponding author under a reasonable request.
